# The effects of genotype on inflammatory response in hippocampal progenitor cells: A computational approach

**DOI:** 10.1016/j.bbih.2021.100286

**Published:** 2021-06-15

**Authors:** Hyunah Lee, Amelie Metz, Amina McDiarmid, Alish Palmos, Sang H. Lee, Charles J. Curtis, Hamel Patel, Stephen J. Newhouse, Sandrine Thuret

**Affiliations:** a Department of Basic and Clinical Neuroscience, Institute of Psychiatry, Psychology and Neuroscience, King's College London, London, United Kingdom; b Department of Biostatistics and Health Informatics, Institute of Psychiatry, Psychology and Neuroscience, King's College London, London, United Kingdom; c Social Genetic & Developmental Psychiatry Centre, Institute of Psychiatry, Psychology and Neuroscience, King's College London, London, United Kingdom; d NIHR BioResource Centre Maudsley, NIHR Maudsley Biomedical Research Centre (BRC) at South London and Maudsley NHS Foundation Trust (SLaM) & Institute of Psychiatry, Psychology and Neuroscience, King's College London, London, United Kingdom; e Health Data Research UK London, University College London, 222 Euston Road, London, United Kingdom; f Institute of Health Informatics, University College London, 222 Euston Road, London, United Kingdom; g The National Institute for Health Research University College London Hospitals Biomedical Research Centre, University College London, 222 Euston Road, London, United Kingdom; h Department of Neurology, University Hospital Carl Gustav Carus, Technische Universität Dresden, Dresden, Germany

**Keywords:** Inflammation, Neural stem cells, Neurogenesis, Hippocampus, Gene variants, In vitro model, Single nucleotide polymorphisms SNP, eQTL

## Abstract

Cell culture models are valuable tools to study biological mechanisms underlying health and disease in a controlled environment. Although their genotype influences their phenotype, subtle genetic variations in cell lines are rarely characterised and taken into account for in vitro studies. To investigate how the genetic makeup of a cell line might affect the cellular response to inflammation, we characterised the single nucleotide variants (SNPs) relevant to inflammation-related genes in an established hippocampal progenitor cell line (HPC0A07/03C) that is frequently used as an in vitro model for hippocampal neurogenesis (HN). SNPs were identified using a genotyping array, and genes associated with chronic inflammatory and neuroinflammatory response gene ontology terms were retrieved using the AmiGO application. SNPs associated with these genes were then extracted from the genotyping dataset, for which a literature search was conducted, yielding relevant research articles for a total of 17 SNPs. Of these variants, 10 were found to potentially affect hippocampal neurogenesis whereby a majority (n=7) is likely to reduce neurogenesis under inflammatory conditions. Taken together, the existing literature seems to suggest that all stages of hippocampal neurogenesis could be negatively affected due to the genetic makeup in HPC0A07/03C cells under inflammation. Additional experiments will be needed to validate these specific findings in a laboratory setting. However, this computational approach already confirms that in vitro studies in general should control for cell lines subtle genetic variations which could mask or exacerbate findings.

## Highlights

• Cell lines are valuable tools to study mechanisms of health and disease.
• Subtle genotypic variations of cell lines are rarely taken into account.
• Genotyping array was performed on a human hippocampal progenitor cell line.
• HPC0A07/03C cell line contains SNPs that may reduce neurogenesis under inflammation.
• In vitro studies should control for cell lines genetic variations.

## 1 Introduction

Cell culture models are routinely used in biomedical research to explore cellular mechanisms underlying health and disease in a controlled environment. As genetic composition impacts the cells’ phenotype, major genetic aberrations are typically controlled for in the cell lines investigated in studies. In contrast, subtle genetic variations are often not considered, even though there is evidence that, for instance, single-nucleotide polymorphisms (SNPs), which are single base substitutions with the rarer allele having a frequency of at least 1% in a population (Keats and Sherman, 2013), can be associated with altered functioning in genetic pathways or with diseases. Previous research (Tokarz et al., 2016) has highlighted the need to characterize the genetic background of cell models in detail in order to fully capture potential consequences on their utility as a model system.

### 1.1 Inflammatory response

Inflammation describes the response of the innate immune systems to challenges caused by injury or disease. Within the central nervous system, this response, also referred to as neuroinflammatory response, is predominantly mediated through the release of pro-inflammatory cytokines, like tumour necrosis factor α (TNFα) and interleukin-1β (IL-1β), chemokines such as CCL5, and reactive oxygen species (Shabab et al., 2017). These mediators are mainly produced by reactive microglia and astrocytes (Salter and Beggs, 2014) but other cells in the brain as well as peripherally derived immune cells can also contribute to a pro-inflammatory environment (Ransohoff and Brown, 2012). Neuroinflammation can have diverse effects on resident cells whereby the intensity and duration of inflammation determine if the consequences are rather protective or destructive. Transient immune activation may enhance neuroplasticity, protect neurons and support tissue repair whereas high levels and chronicity of inflammation can lead to neuronal damage or cell death (for a review see DiSabato et al., 2016).

Inflammation has been found to be involved in many disorders of the central nervous system. Chronic inflammation in particular seems to contribute to neurotoxic effects in neurodegenerative and psychiatric conditions (Stephenson et al., 2018). Thus, the inflammatory response is a key factor of the cellular mechanisms of disease and consequently a relevant process in cell culture models. The genotype of a cell line co-determines the specific reaction of a cell line to an inflammatory stimulus as it can determine the balance between pro- and anti-inflammatory networks. For instance, if pro-inflammatory variants are overrepresented in the genome of a cell line, the cells will exhibit a more intense inflammatory response and may be more susceptible to certain diseases, whereas a higher frequency of anti-inflammatory polymorphisms might have protective effects against inflammatory challenges (Tokarz et al., 2016). Therefore, it is valuable for researchers to be aware of variation in inflammation-related genes in their cell models.

### 1.2 Adult hippocampal neurogenesis

One process in the brain that is known to be affected by chronic inflammation is adult hippocampal neurogenesis. This refers to the capacity of mammalian brains to generate new neurons which persists throughout adulthood, predominantly in the dentate gyrus of the hippocampus (Kempermann et al., 2018). Adult hippocampal neurogenesis takes places in a neurogenic niche called the sub-granular zone which provides a local microenvironment that permits and supports the generation of neurons. This niche contains a pool of neural stem cells (NSCs) that retain the ability to self-renew and differentiate into a range of closely related cell types (Aimone et al., 2014). During the formation of mature neurons, NSCs undergo consecutive developmental stages whereby they first differentiate into specific neural progenitor cells (NPCs) and then into immature neurons before they are integrated into the existing circuitry as mature neurons (Kempermann et al., 2015; Gonçalves et al., 2016). The rate of neurogenesis and the cell fate which can turn towards either neurogenesis or astrogliogenesis are regulated by other cells in the sub-granular zone and by circulating factors from the vasculature (Aimone et al., 2014).

Newborn neurons have been implicated in several hippocampus-dependent cognitive abilities. They are involved in learning in the context of spatial contextual navigation and temporal-based associations. Adult hippocampal neurogenesis also promotes memory function by enhancing pattern separation and thereby reducing memory interference. Newborn neurons also aid memory clearance which is essential for both cognitive flexibility and stable memory consolidation (Deng et al., 2010; Aimone et al., 2014; Miller and Sahay, 2019).

The level of neurogenesis is influenced by various environmental factors, including age (Kuhn et al., 2018), physical exercise (Ma et al., 2017a), and diet (Murphy et al., 2014). Similarly, any pathology affecting the brain, like neurodegenerative disorders or acute injuries (Toda et al., 2019), can impair the generation, differentiation, or survival of new neurons. In addition, adult neurogenesis has been attributed a role in psychiatric disorders such as Major Depressive Disorder (MDD) and Schizophrenia (Kang et al., 2016; Anacker and Hen, 2017). One mechanism which has been proposed to mediate the detrimental effects of these chronic diseases on adult neurogenesis is the concomitant increase of inflammation which has been shown to reduce neurogenesis despite having some neuroprotective properties (Borsini et al., 2015; Miranda et al., 2017). Moreover, evidence suggests that chronic peripheral inflammation causes disturbances in hippocampal neurogenesis and thereby leads to behavioural deficits (Chesnokova et al., 2016).

### 1.3 Aim of investigation

It becomes evident that there is a need to characterize the genotype of cell lines used as a model, especially regarding inflammatory networks, in order to avoid unwanted confounding factors and to ensure that the model is appropriate for the aims of the respective study. Since adult hippocampal neurogenesis is significantly affected by inflammatory processes both in the brain and in the periphery, this also applies to models involving neural stem/progenitor cells.

In this study, the genotype of an established human hippocampal progenitor cell (HPC) line (HPC0A07/03C) was investigated. HPC0A07/03C had been genetically immortalised as neural stem/progenitor cells (NSPCs) upon derivation from a 12-week-old human foetus, and it can be maintained and allowed to proliferate as NSPCs in the presence of growth factors and 4-hydroxytamoxifen. The removal of these factors results in spontaneous differentiation into neuronal and glial cell types (more details described in Materials and Methods), closely mimicking the in vivo processes of human hippocampal neurogenesis. Such stage-specific investigation of NSPCs and the differentiated cell types have been proven to be particularly useful in the context of modelling human hippocampal neurogenesis in vitro (Powell et al., 2017; e.g. Lucia et al., 2020; Smeeth et al., 2020). Therefore, the cell line has been used in several independent studies to study the effects of environmental factors, such as inflammation, on human hippocampal neurogenesis (Zunszain et al., 2012; Borsini et al., 2018, 2020). SNPs located on genes that are associated with chronic or neuroinflammatory response were identified in the genome of HPC0A07/03C, and the existing literature was consulted to determine if they were found to have an effect on the cells’ phenotype. The aim was to investigate how the genetic composition of HPC0A07/03C may affect its response to chronic inflammation, so that this information could be taken into account in studies using this cell line as an experimental model.

## 2 Materials and methods

### 2.1 Cell line and culture conditions

The human hippocampal progenitor/stem cell line HPC0A07/03C (provided by ReNeuron Ltd., Surrey, UK) was used which was derived from hippocampal tissue of a 12-week-old foetus following medical termination. The HPC0A07/03C cells are multipotent and were conditionally immortalised with c-myc-ER technology. Introduction of the c-myc-ER transgene enables the cells to remain in their undifferentiated state and proliferate indefinitely in the presence of epidermal growth factor (EGF), fibroblast growth factor (FGF2), and 4-Hydroxytamoxifen (4-OHT) (Pollock et al., 2006). Removal of these factors terminates proliferation and induces differentiation of the cells into neurons, astrocytes, and oligodendrocytes (Anacker et al., 2011).

DNA from the HPC0A07/03C cells (passage number 20) was isolated from proliferating HPCs 4 days after seeding using TRIzol (Thermo Fisher Scientific, UK) according to the manufacturer's instructions. RNase digestion was carried out using RNase A (Qiagen, Germany). A total of 250 ​ng genomic DNA (5 ​μL of 50 ​ng/μL) was used. The sample had a 260/280 ratio of between 1.7 and 2, as measured using the NanoDrop One (Thermo Fisher Scientific, UK).

### 2.2 Genotyping array

The DNA sample was genotyped at the Institute of Psychiatry, Psychology & Neuroscience (IoPPN) Genomics & Biomarker Core Facility at King's College London using the Infinium OmniExpressExome-8 v1.4 array (Illumina Inc., San Diego, CA, USA), designed to the Genome Reference Consortium Human Reference 37 (GRCh37) assembly. Genotype calls were converted using GenomeStudio and exported into a vcf file using the PLINK software (v2.00, Purcell et al., 2007). A total of 949,081 variants were identified in the genotyping array.

### 2.3 Preparation of variant lists

**Fig. 1:**
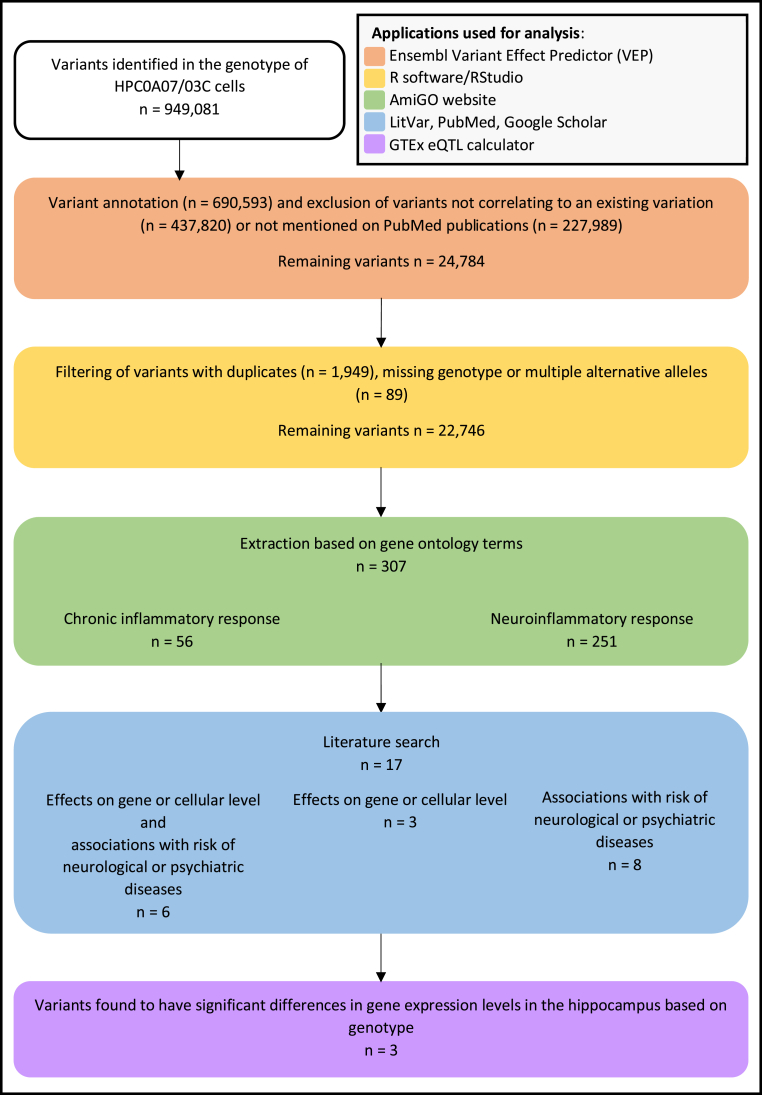
Flow chart summarising the criteria applied during the filtering and extraction of variants expressed in the HPC0A07/03C cell line as well as the inclusion criteria for publications mentioning the resulting variants.

Fig. 1 gives an overview of the analysis undertaken in this study.

In order to select variants for further literature analysis, the list of variants was filtered using the Ensembl Variant Effect Predictor (VEP) (McLaren et al., 2016). Of the variants that could be analysed in this tool (n ​= ​690,593), variants were filtered to remove all variants that did not match an already existing variant in the reference database (n ​= ​437,820) as well as those that are not yet mentioned in any publications on PubMed (n ​= ​227,989). From the remaining 24,784 variants, duplicated variants (n ​= ​1949) as well as SNPs which could not be genotyped or were indicated to have more than one possible alternative allele (n ​= ​89) were removed using the software R (v4.0.0, R Core Team, 2020) and RStudio (v1.2.5042, RStudio Team, 2020). Thus, 22,746 SNPs remained after the filtering process. The R scripts for the filtering process are provided in Supplementary Materials 1.1 and 1.2.

Subsequently, variants were extracted based on gene ontology annotations. To this end, the AmiGO application (Carbon et al., 2009) was used to browse biological processes related to the search terms “inflammation” or “inflammatory”. It was decided to focus on the genes annotated with the gene ontology terms (GOterms) “chronic inflammatory response” and “neuroinflammatory response” as the purpose of this study was to investigate the effect of chronic inflammation in neural progenitor cells. SNPs in the HPC0A07/03C genotyping array that are located on the genes reported by AmiGO were then extracted based on their gene label in R (Supplementary Materials 1.1 and 1.3). For genes associated with the GOterm “chronic inflammatory response”, 56 variants were found in the genotyping array data (Supplementary Materials 1.3.1), whereas the GOterm “neuroinflammatory response” yielded a result of 251 variants (Supplementary Materials 1.3.2). Thus, a total of 307 SNPs were extracted from 22,746 SNPs that remained after the filtering process described above. Only genes expressed in humans were chosen (a full list of relevant genes can be found in the Supplementary Materials 2).

Since this study investigated cells which are formed during the process of neurogenesis, the list of genes associated with the GOterm “neurogenesis” was downloaded from the AmiGO application as well. Of the previously extracted variants, all which are located on genes found in this list (n ​= ​166) (Supplementary Materials 1.3.3) were marked as being related to both inflammation and neurogenesis—the two main biological processes of interest in this study. The downstream analysis was not limited to 166 SNPs and rather included all 307 extracted variants to gather as much information as possible from the existing literature.

### 2.4 Literature research

To investigate what the biological meaning of the selected variants might be, the existing literature was consulted. To this end, primarily the search engine LitVar (Allot et al., 2018) was employed. Here, each individual SNP ID was used as a search term and the results were inspected manually based on cellular mechanisms or disease associations mentioned in the text. In addition, the variant IDs were searched on Google Scholar and PubMed in combination with the search terms “gene expression”, “[respective gene label] expression”, “neural stem cells”, “stem cells”, or “hippocampus”. Results were again inspected manually according to the same criteria.

Publications were included if they reported an effect of the respective SNP in combination with the genotype found in the HPC0A07/03C cell line either on gene or cellular level, for instance alterations in gene expression levels, or on the risk of developing neurological or psychiatric diseases, such as Dementia, Parkinson's Disease or MDD. Consequently, variants that yielded such papers in the literature search were stratified into the following three groups:

A) Previous research on the variant demonstrated effects on gene or cellular level as well as associations with the risk of neurological or psychiatric diseases.
B) Previous research on the variant demonstrated effects on gene or cellular level.
C) Previous research on the variant demonstrated associations with the risk of neurological or psychiatric diseases.

### 2.5 eQTL calculator analysis

The extracted variants for which relevant studies could be retrieved were analysed using the eQTL (expression Quantitative Trait Locus) calculator on the GTEx (Genotype-Tissue Expression project) website (The GTEx Consortium, 2013). The chosen online tool provides access to a database which collects previously computed eQTL data so that statistical information on how specific variants alter gene expression levels in the human tissue of interest can be downloaded. In line with the objective of this project, eQTL values for hippocampal tissue for the selected variants were extracted from the database.

## 3 Results

### 3.1 Findings from the literature search

The AmiGO application revealed that genes associated with the GOterms “chronic inflammatory response” (Supplementary Table 2.1) and “neuroinflammatory response” (Supplementary Table 2.2) were inclusive of the genes associated with both “neurogenesis” and “chronic inflammatory response” and “neuroinflammatory response” (Supplementary Table 2.3). To gather as much information as possible from the existing literature, we decided not to focus exclusively on the “neurogenesis”-associated genes but include all genes associated with “chronic inflammatory response” and “neuroinflammatory response” in the literature search.

The literature search results on the variants associated with the genes identified by AmiGO are summarized in Table 1. A summary of the genotyping array dataset relevant to these genes are presented in Supplementary Table 3.1 and 3.2. Relevant publications that conform to the mentioned inclusion criteria were available on 17 variants. Of these, information on both effects on gene or cellular level and associations with the susceptibility to neurological or psychiatric diseases could be found for 6 SNPs. Effects on gene or cellular level, without indications of an altered risk of diseases, were reported for 3 variants. 8 SNPs have been shown to affect the risk of different neurological or psychiatric diseases, but their cellular or genetic mechanisms have not been uncovered yet. In the following, the relevant findings as well as their interpretation in the context of inflammatory stimulation of the investigated cell line are described in more detail.

**Table 1:** Summary of the results of the literature search on the selected variants found in the genotype of the HPC0A07/03C cell line. Variants marked with an asterisk (∗) were excluded from further discussion as the available information on biological consequences of these SNPs is not sufficient to make valid inferences on the phenotype of HPC cells with this genotype.

Variant ID	Gene	Gene ontology term	Genotype in HPC cell line	Publication	Summary of the findings related to the variant
					
A) Variants with demonstrated effects on gene or cellular level as well as associations with the risk of neurological or psychiatric diseases					
rs10108662	IDO1	Chronic inflammatory response	CA (heterozygous)	Duan et al. (2019)	A allele was associated with decreased incidence of postpartum depressive symptoms, potentially due to lower perinatal plasma kynurenine levels and a lower kynurenine/tryptophan ratio compared to CC genotype carriers
rs5275	PTGS2 = COX2	Neuroinflammatory response	AG (heterozygous)	Bialek et al. (2020)	AG genotype correlated with increased risk of Major Depressive Disorder in female subjects
				Moore et al. (2012)	C allele was associated with COX2 overexpression by disrupting micro-RNA-mediated mRNA degradation (not replicated by Piranda et al. (2017))
rs1800630 (= -863C/A)	TNF = LTA	Chronic inflammatory response, neurogenesis	CC (homozygous reference)	Chen et al. (2019)	CC genotype was associated with higher serum TNFα levels
				Higuchi et al. (1998)	Transcriptional promoter activity of the A allele was 2.0-fold higher than that of the reference allele in response to concanavalin A stimulation
				Tsutsumi et al. (2007)	C allele was associated with increased risk of Alzheimer's Disease (original paper unavailable, reported in Montgomery et al. (2018))
rs1800629 (= -308G/A)	TNF = LTA	Chronic inflammatory response, neurogenesis	GA (heterozygous)	Review by Barnes et al. (2017)	Literature shows contradicting results on the association between the variant and the occurrence of depression: - A allele increases risk of Major Depressive Disorder (Jun et al., 2003) and post-stroke depression (Kim et al., 2012) - GG genotype more frequent in subjects with MDD (Clerici et al., 2009), late-life MDD (Cerri et al., 2009), or in oncology patients with depression (Dunn et al., 2013) - No association with depression (Misener et al., 2008; Haastrup et al., 2012; Kim et al., 2013; Tartter et al., 2015) - A allele associated with labile anger and fatigue, but not depression, in IFNα-induced depression (Lotrich et al., 2010, 2013)
				Baune et al. (2012)	A allele was associated with decreased hippocampal grey matter concentration in both hemispheres in healthy subjects
rs1800629 (= -308G/A)				Review by Dittmar and Schuttelaar (2017)	Literature shows conflicting results on whether the variant is functional, as indicated by increased transcriptional activity and/or TNFα production: - A allele increased transcriptional activity (Kroeger et al., 1997; Wilson et al., 1997; Wu and McClain, 1997; Karimi et al., 2009) - A allele upregulated circulating TNFα levels (Stüber et al., 1996; Mira et al., 1999; Das et al., 2006; Oliveira et al., 2016); more recently replicated in Arbab et al. (2017), Ding et al. (2019), González et al. (2003), and Pan et al. (2019) - A allele associated with increased TNFα production following endotoxin stimulation (Louis et al., 1998; Heesen et al., 2003; Das et al., 2006) - No effect (Pociot et al., 1993; He et al., 1995; Kubota et al., 1998; Uglialoro et al., 1998; Bayley et al., 2001; Jong et al., 2002; Kovar et al., 2007; Taudorf et al., 2008)
				Kumari et al. (2018)	GA genotype was associated with higher levels of TNFα and lower levels of IL10 and possibly affected lipid metabolism as reflected by the elevated levels of lipid profile like total cholesterol, triglyceride, and low density lipoprotein in individuals with coronary artery disease
				Review by Oxenkrug (2007)	A allele might contribute to development of vascular cognitive impairment
rs522807	TNFR2	Neuroinflammatory response; neurogenesis	CA (heterozygous)	Fairfax et al. (2011)	Variant increased basal expression of TNFR2 mRNA and was associated with a decreased tolerance for LPS stimulation, as indicated by elevated TNF release following secondary LPS stimulation
rs222747	TRPV1	Neuroinflammatory response	GG (homozygous alternative)	Buttari et al. (2017)	GG genotype increased pain and weakness during flu-like symptoms in Multiple Sclerosis patients
				Deering-Rice et al. (2016)	G allele associated with increased TRPV1 mRNA expression
				Mori et al. (2012)	Subjects with GG genotype exhibited larger short-interval intracortical facilitation explored through paired-pulse TMS of the primary motor cortex, indicating enhanced glutamate transmission
				Stampanoni Bassi et al. (2019)	G allele associated with lower TNFα CSF levels in Multiple Sclerosis patients

B) Variants with demonstrated effects on gene or cellular level					
rs11666254	FPR2	Neuroinflammatory response; neurogenesis	AG (heterozygous)	Zhang et al. (2017a)	Variation associated with lower FPR2/ALX mRNA and protein expression, decreased promoter activity of the FPR2/ALX gene and higher TNFα production from peripheral blood leukocytes following LPS stimulation
rs689470	PTGS2	Neuroinflammatory response	GG (homozygous reference)	Wang et al. (2013)	GG genotype increased PTGS2 mRNA expression levels
rs4790522	TRPV1	Neuroinflammatory response	CC (homozygous alternative)	Zhang et al. (2017b)	Variation caused disappearance of binding site miR-141-3p
					
C) Variants with demonstrated associations with the risk of neurological or psychiatric diseases					
∗ rs17735961	CCL11	Chronic inflammatory response, neurogenesis	CC (homozygous reference)	Kang et al. (2018)	Haplotypes GCT, ACT, and GCC containing rs4795896, rs17735961 and rs17809012 were associated with schizophrenia
∗ rs17809012			AA (homozygous reference)		
∗ rs5930	LDLR	Neuroinflammatory response; neurogenesis	AG (heterozygous)	Oliveira et al. (2017)	In patients with Alzheimer's Disease, rs5930-AG/rs11669576-GG genotypes were associated with less irritability - rs5930: A allele carriers had less delusions when mildly impaired, less anxiety when moderately impaired, and higher apathy and irritability when severely impaired, while G allele carriers had less apathy when mildly and moderately impaired - rs11669576: A allele carriers had less anxiety and more aberrant motor behaviour
∗ rs11669576			GG (homozygous reference)		
∗ rs11079727	MAPT	Neuroinflammatory response; neurogenesis	CA (heterozygous)	Gan-Or et al. (2012)	CA genotype associated with later age of onset in LRRK2-associated Parkinson's Disease patients
rs1467967	MAPT	Neuroinflammatory response; neurogenesis	GA (heterozygous)	Babić Leko et al. (2018)	GA genotype associated with increased t- and p-tau CSF levels in Alzheimer's Disease/Mild Cognitive Impairment patients
∗ rs2292305	THBS1	Chronic inflammatory response	AG (heterozygous)	Lu et al. (2014)	Variant was associated with autism risk whereby G allele seems to have a protective effect
rs1061624	TNFR2	Neuroinflammatory response; neurogenesis	AG (heterozygous)	Stacey et al. (2017)	A allele was associated with decreased hippocampal grey matter volume, compared to GG genotype
				Suchanek-Raif et al. (2018)	GA genotype associated with higher risk of schizophrenia among individuals with a family history of SZ

6 SNPs are not discussed here as the information on biological consequences of these variations in the available literature is too little to create a valid link to the phenotype that HPCs might display under inflammatory conditions. These SNPs include rs17735961 and rs17809012 of the CCL11 gene as they have only been assessed in the context of a haplotype (Kang et al., 2018) and the third variant of this haplotype was not found to be expressed in the HPC cell line. rs5930 and rs11669576 of the LDLR gene was also excluded as it would be too speculative to relate a genotype that might alter the severity of specific symptoms in Alzheimer's Disease (Oliveira et al., 2017) to neurogenesis. Similarly, despite the proposed link between neuropsychiatric disorders and dysregulation of adult hippocampal neurogenesis (Ilieva et al., 2018; Sacco et al., 2018; Vitrac and Cloëz-Tayarani, 2018), it was deemed too tentative to suggest that the protective effect of rs2292305 (THBS1 gene, Lu et al. (2014)) against autism spectrum disorders can be extended to the phenotype of HPCs. Accordingly, rs11079727 on the MAPT gene was also excluded from further discussion because the response of HPCs to inflammation can hardly be deduced from the existing literature. Although CA heterozygous genotype seems to be associated with later onset of Parkinson's Disease (Gan-Or et al., 2012), no evidence regarding inflammation was reported for this association. The most relevant finding that demonstrated a link between hippocampal neurogenesis and Parkinsons' Disease could be found in studies that showed a partial contribution of deficient hippocampal neurogenesis to non-motor symptoms of Parkinson's Disease (Regensburger et al., 2014; Lim et al., 2018) while being independent from the underlying degeneration of dopaminergic neurons (Ermine et al., 2018). However, these studies did not examine whether inflammation plays a role.

## 4 Variants with demonstrated effects on gene or cellular level as well as associations with the risk of neurological or psychiatric diseases

### 4.1 rs10108662 (IDO1)

The polymorphism rs10108662 is located on the indoleamine 2,3-dioxygenase 1 (IDO1) gene. The enzyme IDO plays a major role in the first step of the kynurenine (KYN) pathway in that it catalyses the conversion of tryptophan (TRP) into KYN which is a precursor to both quinolinic acid and kynurenic acid. While quinolinic acid seems to have neurotoxic effects, kynurenic acid is potentially neuroprotective. Inflammatory stimuli, such as interferon-gamma (IFNγ), TNFα, and lipopolysaccharide (LPS), have been shown to upregulate IDO, in turn increasing activity of the KYN pathway (Zunszain et al., 2012; Jones et al., 2013).

The HPC0A07/03C cell line carries the CA genotype on rs10108662. Presence of the A allele has been associated with decreased KYN plasma levels and, consequently, a lower KYN/TRP ratio (Duan et al., 2019). The latter is considered to indicate decreased IDO activity but there is no direct evidence yet on whether variation in rs10108662 alters IDO enzymatic activity and what the underlying mechanisms could be.

Inhibition of IDO1 protein activity has previously been reported to exert a neuroprotective influence on human neural stem cells under IFNγ challenge (Chen et al., 2012). Accordingly, IFNγ has been shown to inhibit proliferation of mesenchymal stem cells by activating IDO (Croitoru-Lamoury et al., 2011). Considering that the alternative allele on IDO rs10108662 might decrease IDO activity, these results suggest that cells with this genotype are somewhat protected from neurotoxicity under inflammation. This would also be in keeping with the finding that A allele carriers have a lower risk for developing postpartum depressive symptoms (Duan et al., 2019). Interestingly, Zunszain et al. (2012) previously investigated the response of another HPC0A07/03C cell line to IL-1β stimulation and focused on effects within the KYN pathway. IL-1β was found to induce an overexpression of IDO and KYN as well as increased levels of enzymes involved in the production of quinolinic acid which is thought to be neurotoxic. Consequently, neurogenesis was reduced, as indicated by lower numbers of doublecortin-positive neuroblasts and mature, microtubule-associated protein-2-positive neurons. Therefore, despite the potentially neuroprotective genotype on the IDO1 gene, inflammation still has a profound negative impact on neurogenesis in the HPC cell line. However, the single study specifically on this cell line does not exclude the possibility that the IDO1 genotype attenuated the inflammatory response of the cells to some extent. Also, the response of the cells might depend on the pro-inflammatory stimulus and thus, IFNγ treatment might lead to different results.

### 4.2 rs5275 and rs689470 (PTGS2)

In the genotyping array, two variants on the Prostaglandin G/H Synthase 2 (PTGS2), also referred to as COX2, were identified and will be discussed together here, although they were stratified into different groups based on their literature search results. Firstly, the HPC cell line was found to be homozygous for the G allele on rs689470. This genotype has been associated with augmented cyclooxygenase-2 (COX2) mRNA expression (Wang et al., 2013). For the COX2 variant rs5275, the HPC cell line carries the heterozygous genotype (AG). Existing literature on this variation has found an upregulation of COX2 expression with the G allele (Moore et al., 2012), although this finding was not replicated by Piranda et al. (2017). Furthermore, an increased risk of MDD occurrence in females with the AG genotype has been reported (Bialek et al., 2020) which is relevant for the HPC0A07/03C cell line since it was derived from a female donor.

The enzyme COX2 is involved in the biosynthesis of prostaglandins and seems to play an important role in adult hippocampal neurogenesis both in health and disease. Genetic and pharmacological inhibition of COX2 has been found to disturb maintenance of the NSC population, cell proliferation, and cell differentiation in the dentate gyrus (Nam et al., 2013, 2015). Also, injury-induced proliferation of neural progenitor cells was attenuated by COX2 deficiency (Sasaki et al., 2003; Jung et al., 2006), suggesting that COX2 modulates the rate of adult neurogenesis.

Increased levels of COX2 in the hippocampus have been associated with diminished neurogenesis in the context of acute inflammation caused by LPS. Ma et al. (2017b) reported that COX2 mRNA expression levels in the dentate gyrus of rats were enhanced 3 ​h after LPS injection, before returning to basal levels. While LPS stimulation was sufficient to decrease NSC proliferation, simultaneous inhibition of COX2 led to a further reduction of numbers of Ki67- and Bromodeoxyuridine- (BrdU) positive cells after 24 ​h, suggesting a protective effect of COX2 on neurogenesis under inflammation. This is supported by Watanabe et al. (2017) who found that overexpression of COX2 in rat granule cells facilitated cell proliferation and neuronal plasticity in the granule cell layer following exposure to N-methyl-N-nitrosourea (MNU). Conversely, Bastos et al. (2008) provided evidence that COX2 mediates the attenuating effect of 10.13039/501100012274LPS on hippocampal neurogenesis. In their study, the LPS-induced impairment to neurogenesis was reversed by injecting COX and COX2-seletive inhibitors which indicates that COX2 is involved in the negative impact of LPS. Furthermore, as the number of BrdU-labelled cells was reduced 7 and 21 days, but not 2 ​h, after BrdU injection, the authors concluded that the survival of newborn cells, rather than cell proliferation, was affected by LPS.

Overall, research shows contrasting results on the consequences of COX2 upregulation for NSCs as both protective effects during the proliferative stage as well as impairment of newborn cell survival have been described. Moreover, it should be kept in mind that there is no consensus on whether the COX2-related genotype in the HPC cell line investigated here is in fact associated with increased COX2 expression. The finding that heterozygosity of rs5275 is linked to an increased susceptibility to MDD might indicate negative consequences of this genotype on adult hippocampal neurogenesis as dysfunctional neurogenesis has been implicated in the pathophysiology of MDD (Vaidya et al., 2007; Micheli et al., 2018).

### 4.3 rs1800630 (TNFα)

The genotyping array identified two polymorphisms on the TNF gene. For the first one, rs1800630, the HPC0A07/03C cells are homozygous for the reference allele (CC). This genotype has been related both to higher serum TNFα levels in response to toxic metal exposure (Chen et al., 2019) and to decreased transcriptional promoter activity following concanavalin A exposure (Higuchi et al., 1998). Furthermore, the C allele of this SNP has been implicated in increased risk of AD (Tsutsumi et al., 2007).

The contrary consequences for TNFα production that were observed for rs1800630 suggest that different stimuli trigger different responses in this genotype. As inhibitory effects were found following a single inflammatory challenge, whereas a pro-inflammatory response was reported in a long-term toxic environment, this could indicate that the genotype has a negative influence under chronic inflammation. This would fit the association with AD occurrence which tied to neuroinflammation and, thereby, to high levels of TNFα.

TNFα is a pro-inflammatory cytokine that is primarily secreted by macrophages but can also be released by reactive microglia and other cells of the CNS in response to immune activation. It has been proposed to underlie chronic inflammation in various pathological conditions, and particularly in neurodegenerative diseases (Probert, 2015; Miranda et al., 2017). TNFα mediates anti-neurogenic effects of inflammatory environments by suppressing proliferation of hippocampal NSCs, by promoting apoptosis of progenitor cells, and by driving differentiating cells towards astrogliogenesis, rather than towards a neuronal fate (for reviews, see Borsini et al. (2015), Kohman and Rhodes (2013), and Miranda et al. (2017)).

This evidence points towards a neurotoxic tendency exerted by TNF rs1800630. Possibly, the HPC0A07/03C cells’ genotype does not affect their response to a short-term inflammatory challenge, but the findings indicate that the variant leads to increased TNFα production under chronic inflammation which intensifies detrimental effects on hippocampal neurogenesis.

### 4.4 rs1800629 (TNFα)

For the second variant of the TNF gene in this dataset, the HPC0A07/03C cells carry the heterozygous genotype (GA). Rs1800629 has been widely investigated and implicated in various diseases (El-Tahan et al., 2016) but results are often discrepant.

Firstly, as summarized by Dittmar and Schuttelaar (2017), the A allele of this SNP has been found to promote transcriptional activity and increase TNFα expression in many experiments, while others failed to reproduce this effect. Overall, the consensus seems to be that variation on rs1800629 leads to increased production of TNFα. As described above, this would exacerbate inhibition of adult neurogenesis in chronic inflammatory conditions.

Secondly, Kumari et al. (2018) reported lower levels of IL10 in subjects with the GA genotype. IL10 is an anti-inflammatory cytokine and has been proposed to attenuate inflammation and resulting tissue damage (Ouyang et al., 2011). Still, lower IL10 levels could be protective for HPCs, considering that IL10 has been found to impair neurogenesis in the subventricular zone by locking progenitor cells in their undifferentiated state (Perez-Asensio et al., 2013; Pereira et al., 2015).

Thirdly, the SNP may alter lipid metabolism as heterozygosity on this position has been associated with elevated cholesterol, triglyceride and low-density lipoprotein (LDL) levels (Kumari et al., 2018). Although blood lipids seem to be essential for regulation cell metabolism under inflammation (Zhang et al., 2018) as well as for neurogenesis (Knobloch and Jessberger, 2017), excessive amounts can impair hippocampus-dependent cognition, for instance in the context of a high-cholesterol diet (Ledreux et al., 2016). The specific impact of lipids on HPCs remains to be investigated, however.

Regarding phenotype, there is no clear evidence whether a link between rs1800629 and diverse manifestations of depressive disorders exists (reviewed by Barnes et al. (2017)). Oxenkrug (2007) argues that variation on rs1800629 is involved in Vascular Cognitive Impairment by activating IDO gene transcription. Both depression and Vascular Cognitive Impairment, a form of dementia, are pathologies associated with chronic inflammation and impaired adult neurogenesis (Steiner et al., 2006), suggesting that the genotype promotes inflammatory conditions and negatively impacts HPC0A07/03C cells. As discussed in section ‘1.1 rs10108662 (IDO1)’, increased IDO activity also reduces neurogenesis. In addition, Baune et al. (2012) reported lower hippocampal grey matter density in subjects with the GA genotype. Depression seems to be linked to decreased hippocampal volume (Videbech and Ravnkilde, 2004), supporting the notion that the genotype is implicated in the occurrence of MDD. Yet, hippocampal grey matter density or volume is not indicative of the functionality of adult neurogenesis, as will be discussed below (see section ‘3.2 rs1061624 (TNFR2)).

Taken together, there is considerable evidence that variation on rs1800629 in the HPC0A07/03C cell line's genotype could facilitate inflammation due to increased TNFα and decreased IL10 expression, thereby putting the cells at risk for pathological alterations associated with psychiatric and neurodegenerative diseases and potentially impairing hippocampal neurogenesis. It must be kept in mind, however, that most of the experimental findings are still controversial and need to be investigated further.

### 4.5 rs522807 (TNFR2)

For rs522807, the HPC0A07/03C cell line carries the heterozygous genotype. This genotype has been linked to elevated Tumour necrosis factor receptor 2 (TNFR2) mRNA expression and increased TNFα production following LPS challenge (Fairfax et al., 2011).

TNFR2-mediated TNFα signalling plays a crucial role in attenuating inflammation (Peschon et al., 1998). TNFR2 expression is upregulated by TNFα in autoimmune diseases, possibly as a compensatory mechanism (Faustman and Davis, 2013). It is considered to be neuroprotective in that it suppresses neuronal cell death, for instance, by upregulating anti-apoptotic genes in oligodendrocyte progenitor cells exposed to oxidative stress or in a model of Multiple Sclerosis (MS) (Maier et al., 2013; Madsen et al., 2016) and by activating the PI3K-PKB/Akt pathway in a model of Parkinson's Disease (Fischer et al., 2011). TNFR2 has also been found to protect neurons from glutamate-induced excitotoxicity (Marchetti et al., 2004; Dolga et al., 2008). In the hippocampus specifically, experiments showed that TNFR2 reduces Aβ-induced TNFα levels (Detrait et al., 2014) and limits AMPA-related excitotoxicity (Bernardino et al., 2005). Regarding neurogenesis, Chen and Palmer (2013) reported that TNFR2 enhances the baseline rate of neurogenesis, in particular the growth and survival of hippocampal NSCs, and inhibits the decline of neurogenesis in an irradiation-induced model of chronic inflammation.

Taken together, these findings point towards a protective effect of increased TNFR2 expression on HPCs. Due to their genotype, the HPC0A07/03C cells may be more resistant to inflammatory challenge, especially during maturation and survival phases. This is only contradicted by the second finding of Fairfax et al. (2011) that rs522807 leads to lower endotoxin tolerance, as evidenced by heightened TNFα production following repeated recurrent LPS stimulation. This could indicate that the initial beneficial influence of the genotype subsides when inflammatory stimuli are repeated as, potentially, TNFR2 cannot compensate the increasing TNFα load anymore.

### 4.6 rs222747 (TRPV1)

On the Transient Receptor Potential Vanilloid Type 1 (TRPV1) gene, the cells carry the minor allele (G) for rs222747. This allele has been associated with elevated expression of TRPV1 (Deering-Rice et al., 2016) and with enhanced glutamate transmission (Mori et al., 2012). It also has been related to lower TNFα CSF levels (Stampanoni Bassi et al., 2019) and increased severity of specific symptoms (Buttari et al., 2017) in patients with MS.

TRPV1 is a capsaicin receptor and possibly regulates neurogenesis (Ramírez-Barrantes et al., 2016). While TRPV1 deficiency has resulted in increased proliferation of NSCs, even under chronic unpredictable stress (Stock et al., 2014; Wang et al., 2017), activation of TRPV1 through capsaicin seems to deteriorate proliferation of progenitor cells in the SGZ as well as survival of newborn neurons in the dentate gyrus in vivo, possibly mediated via Notch and Hedgehog/Wnt signalling pathways (Stock et al., 2014; Wang et al., 2017, 2018). Hence, overexpression of TRPV1 could decrease the resilience of HPCs to stress and make them more susceptible to inflammatory environments.

Glutamate is the major excitatory neurotransmitter in the brain and is involved in neuroinflammatory responses (reviewed in Dantzer and Walker (2014), Haroon et al. (2017) and McNally et al. (2008)). Although glutamate evidently is also essential for functional neurogenesis and might support neuronal regeneration after injury, excessive levels of glutamate are proposedly neurotoxic and might play a role in the aetiology of depression. The existing literature suggests that glutamate can both promote or diminish proliferation, differentiation, survival, and migration of neural progenitor cells, depending on the underlying mechanism, environment and the specific glutamate receptor involved (Schlett, 2006; Jansson and Åkerman, 2014; Rubio-Casillas and Fernández-Guasti, 2016). Therefore, it is challenging to form a comprehensive hypothesis of how, in general, increased glutamate transmission caused by genetic variation might alter the HPC0A07/03C cell line's response to inflammatory challenge.

Nevertheless, evidence suggests that excessive glutamate associated with the GG genotype of rs222747 is implicated in aggravating the pathophysiology of MS, a chronic inflammatory and demyelinating disease. While the GG genotype of rs222747 is linked to enhanced glutamate transmission in the brain (Mori et al., 2012), MS patients carrying this genotype are known to display exacerbated symptoms (Buttari et al., 2017), even though the same SNP seems to reduce TNFα cytokine expression in the CSF which has been proposed to alleviate the disease (Taoufik et al., 2011). Furthermore, insights from mouse models of MS suggest that while the inflammatory environment of the brain may enhance proliferation of HPCs during the acute phase (Giannakopoulou et al., 2013, 2017), the number of proliferating cells and immature neurons is likely to fall as the disease progresses (Guo et al., 2010; Khodanovich et al., 2019; Zhang et al., 2020). Taken together, the GG genotype of rs222747 is more likely to contribute to the vulnerability of HPC0A07/03C cells to excitotoxicity generated by elevated glutamate levels, despite reduced TNFα production due to the same genotype. Therefore, the neurogenic capacity is expected to be diminished in chronic inflammatory environments.

## 5 Variants with demonstrated effects on gene or cellular level

### 5.1 rs11666254 (FPR2)

For rs11666254 of the formylpeptide receptor 2 (FPR2) gene a heterozygous genotype (AG) was identified in the HPC0A07/03C cell line. Zhang et al. (2017a) reported that the rs11666254 polymorphism was associated with a reduction in FPR2 mRNA and protein expression.

FPR2 can have both pro- and anti-inflammatory effects, depending on the activating ligand, as reviewed by Corminboeuf and Leroy (2015) and Trojan et al. (2020). Accordingly, research has found varying functions of FPR2 for NSCs in different environments. On the one hand, in a brain injury model FPR1/2 seem to support the migration of transplanted NSCs through F-actin polymerization and to promote neuronal fate mediated via ROS and PI3K-AKT signalling pathways (Wang et al., 2016; Zhang et al., 2017c). On the other hand, Aβ-induced accelerated senescence of NSPCs has been found to be mediated via FPR2-dependent activation of ROS-p38 MAPK signalling as FPR2 was overexpressed in the dentate gyrus of APP/PS1 transgenic mice (He et al., 2013). Moreover, FPR2/3 knockout in a mouse model of depression ameliorated anxiety and depression-like behaviour, while limiting neuronal death in the hippocampus (Peritore et al., 2020). Thus, despite some beneficial influence on neurogenesis, FPR2 might exacerbate the impairment of NSCs under conditions associated with chronic inflammation, such as Alzheimer's Disease and depression. This suggests that the lower basal expression of FPR2 related to the HPC0A07/03C cell line's genetic makeup might be an advantage when the cells are exposed to inflammatory stimuli.

However, in addition to decreased FPR2 expression rs11666254 might also lead to increased expression of the pro-inflammatory cytokine TNFα which, as explained in section ‘1.3 rs1800630 (TNFα)’, promotes further inflammation and impairs adult neurogenesis. Thus, the observed variation on the FPR2 gene in the HPC0A07/03C cells might have both positive and negative biological consequences for HPCs which potentially even each other out.

### 5.2 rs4790522 (TRPV1)

For the second variant found on the TRPV1 gene, HPC0A07/03C cells were homozygous for the alternative allele for rs4790522 and variation on this SNP has been linked to the disappearance of the microRNA binding site miR-141-3p (Zhang et al., 2017b).

Jiang et al. (2017) reported that upregulated expression of miR-141-3p was related to inhibited proliferation, differentiation, and migration of NSCs in response to stimulation with the anaesthetic propofol. Similarly, proliferation of human mesenchymal stem cells was found to be suppressed by miR-141-3p (Qiu and Kassem, 2014).

As the HPC0A07/03C cells might be lacking the binding site for this microRNA due to variation in rs4790522, TRPV1 potentially is less regulated by miR-141-3p in these cells. Whether this is sufficient to protect the cells from the inhibitory effect that miR-141-3p has on neurogenesis remains to be tested.

## 6 Variants with demonstrated associations with the risk of neurological or psychiatric diseases

### 6.1 rs1467967 (MAPT)

On the Microtubule Associated Protein Tau (MAPT) gene, the HPC0A07/03C cells are heterozygous for rs1467967. This genotype has been associated with elevated tau levels in the cerebrospinal fluid (CSF) of patients with Mild Cognitive Impairment or AD (Babić Leko et al., 2018).

While the tau protein is important for various cellular functions, it can also cause neurotoxicity through its hyperphosphorylation and accumulation in neurofibrillary tangles and has been implicated in the pathophysiology of AD, frontotemporal dementia and other tauopathies. It also seems to play a role in neuroinflammation. As reviewed by Laurent et al. (2018), tau pathology is sufficient to induce reactive microgliosis and astrocytosis. On the other hand, neuroinflammation might promote tau hyperphosphorylation and it has been proposed that this process partially initiates disease progression. This dynamic creates a vicious cycle of neurotoxicity. Adult neurogenesis has also been found to be negatively impacted by tau aggregation. In vivo studies showed that tau hyperphosphorylation drives stress-induced decreases of neurogenesis by reducing both the number of BrdU- and DCX-positive cells in the hippocampus (Dioli et al., 2017; Criado-Marrero et al., 2020). Houben et al. (2019) reported that mice expressing mutant tau had reduced granular layer volume and less neuronal precursor and proliferating cells. In all studies, genetic deletion of endogenous tau reversed these effects. Komuro et al. (2015) also showed that expression of human tau in a mouse model of tauopathy led to reduced cell proliferation in the hippocampus.

These results could indicate that due to their genetic predisposition to increased tau production the HPC0A07/03C cells are more prone to developing tau pathology under chronic inflammatory conditions, such as AD pathology, which in turn aggravates the detrimental effects on maintenance of the progenitor cell pool.

### 6.2 rs1061624 (TNFR2)

The cells were found to be heterozygous for the second SNP on the TNFR2 gene, rs1061624. Even though this genotype has been associated with an increased risk of developing Schizophrenia (Suchanek-Raif et al., 2018), this was only true for subjects with a family history of the disease and such information is not available for the donor of the HPC0A07/03C cell line.

Another study reported a link between variation on rs1061624 and a reduction in hippocampal grey matter volume (Stacey et al., 2017). Decreased generation of newborn cells could potentially underlie this phenomenon, indicating a lower baseline rate of adult neurogenesis caused by the cells' genotype. This is supported by the finding that hippocampal volume seems to be affected by conditions related to chronic inflammation and reduced neurogenesis, such as AD and MDD (Kim and Won, 2017; Sheline et al., 2019). However, compared to other factors, the contribution of adult neurogenesis to the size of the hippocampus is relatively small and possibly limited to particular structures within the hippocampus, like the dentate gyrus (Zunszain et al., 2011; Sheline et al., 2019). Accordingly, Stacey et al. (2017) attributed the changes in hippocampal volume associated with variation on the TNFR2 gene to increased cell death since, as discussed for rs522807 above in section ‘1.5 rs522807 (TNFR2)’, TNFR2 modulates apoptotic mechanisms. Therefore, hippocampal volume is not sufficient to draw conclusions on neurogenic processes.

### 6.3 eQTL calculator results

In order to validate the findings in the existing literature the eQTL calculator analysis was added as an additional step to determining which SNPs affect gene expression in the hippocampus. Results of the variant search in the eQTL database are summarized in Table 2. Only 3 SNPs demonstrated a statistically significant difference in gene expression between the genotypes. These are shown in Table 3 and discussed in the following.

**Table 2:** Results of the eQTL calculator analysis on the variants that yielded relevant results in the literature search, downloaded from the GTEx website. P-values indicate whether the variants significantly affect gene expression levels in the hippocampus.

Variant ID	Gene label	p-value	p <0.05
rs1800630	LTA/TNFα	0.021	∗
rs522807	TNFRSF1B/TNFR2	0.021	∗
rs11079727	MAPT	0.045	∗
rs2292305	THBS1	0.070	
rs1800629	LTA/TNFα	0.17	
rs5930	LDLR	0.30	
rs1467967	MAPT	0.36	
rs222747	TRPV1	0.49	
rs5275	PTGS2	0.50	
rs4790522	TRPV1	0.74	
rs1061624	TNFRSF1B/TNFR2	0.90	
rs11669576	LDLR	0.94	
rs689470	PTGS2/COX2	0.94	
rs11666254	FPR2	0.95

**Table 3:** Results and violin plots of the eQTL calculator analysis for variants that yielded relevant results in the literature search which had a p-value below the <0.05 threshold. Statistical values and plots were downloaded from the GTEx website.

Variant ID	Gene name	Genotype in HPC cell line	p-value	eQTL violin plot	Median of the normalized expression in hippocampal tissue		
					**Homozygous reference**	**Heterozygous**	**Homozygous alternative**
rs1800630	TNFα/LTA	CC (homozygous reference)	0.021		-0.007550	0.01510	-0.8813
rs522807	TNFRSF1B/TNFR2	CA (heterozygous)	0.021		-0.007550	-0.03020	0.4884
rs11079727	MAPT	AC (heterozygous)	0.045		0.06044	-0.1516	0.5915

### 6.4 rs1800630 (TNFα)

Data on how rs1800630 affects TNF gene expression levels in the hippocampus taken from the eQTL database were consistent with the previously mentioned findings in the existing literature in that the C allele upregulates TNFα (Chen et al., 2019) in comparison to the homozygous genotype including the alternative allele.

### 6.5 rs522807 (TNFR2)

For rs522807, the eQTL calculator results did not confirm literature findings. While Fairfax et al. (2011) reported enhanced TNFR2 mRNA expression with the heterozygous genotype in human peripheral blood mononuclear cells, the eQTL database suggests that this genotype has lower gene expression levels in the hippocampus compared to the other genotypes. Thus, the neuroprotective effects of increased TNFR2 (Chen and Palmer, 2013; Faustman and Davis, 2013), which have been described in section ‘1.5 rs522807 (TNFR2)’, might not readily apply to HPCs, and further studies will be needed to examine the changes of TNFR2 expression and its effect on inflammatory response directly in HPCs.

### 6.6 rs11079727 (MAPT)

According to the eQTL database, rs11079727 significantly alters MAPT gene expression in the hippocampus whereby heterozygosity is associated with lower levels compared to the homozygous genotypes. Considering the deleterious impact of excess tau protein on adult hippocampal neurogenesis (Komuro et al., 2015; Dioli et al., 2017), this potentially has neuroprotective effects on HPCs. It could also explain why individuals with the CA heterozygous genotype have a later age of onset in Parkinson's Disease (Gan-Or et al., 2012), which is associated with tauopathy.

## 7 Discussion

### 7.1 Summary of the main findings

The aim of this project was to investigate the genotype of an HPC cell line frequently used as an in vitro model of hippocampal neurogenesis and to elucidate how it might affect the cells' response to chronic inflammation. To this end, variants on genes implicated in chronic and neuroinflammatory response were extracted from the genotyping array data of the HPC0A07/03C cell line's DNA and their genetic or cellular effect as well as their association with neurological or psychiatric diseases were determined by analysing the existing literature. To confirm the literature findings, the SNPs' influence on gene expression levels in the hippocampus were inspected in an eQTL database. By analysing the variants' reported consequences for gene expression, cellular mechanisms or disease risk, their potential effects on neurogenesis were inferred.

Overall, 17 polymorphisms met the inclusion criteria and yielded relevant results in the literature search. 6 of those SNPs had to be excluded from further discussion because the findings from previous experiments were not sufficient to derive an evidence-based assumption on their influence on the HPCs’ response to inflammation. In the eQTL calculation, only three variants were found to cause a statistically significant difference in gene expression within the hippocampus between the genotypes. Table 4 gives an overview on how each of the included variants might affect hippocampal neurogenesis, subdivided into the sequential stages of neurogenesis. Taken together, the variants that were found to potentially participate in the regulation of hippocampal neurogenesis based on findings in previous studies seem to have a negative effect on all phases of the neurogenic process.

**Table 4:** Overview of potential effects of the HPC0A07/03C cell line's genotype on the different stages of neurogenesis under chronic inflammation. If not specified otherwise, the results are based on findings in the existing literature. Legend: ↑ increase; ↓ decrease; ​= ​no difference; ? insufficient evidence.

Variant ID	Stages of neurogenesis					Overall effect
	**NSPC self-renewal/survival**	**NSPC proliferation**	**NSPC migration**	**Differentiation of NSPCs into neurons**	**Survival of newborn neurons**	
rs10108662 (IDO1)		↑				↑
rs5275 (PTGS2)		↑?			↓?	↓?
rs1800630 (TNFα) – according to literature search and eQTL results	↓	↓		↓	↓	↓
rs1800629 (TNFα)	↓	↓		↓	↓	↓
rs522807 (TNFR2) – according to literature search	↑					↑
rs522807 – according to eQTL results	↓					↓
rs222747 (TRPV1)		↓			↓	↓
rs11666254 (FPR2)	↓	↓	↓	↓	↑/↓	↓
rs689470 (PTGS2)		↑			↓	=
rs4790522 (TRPV1)		↑?	↑?	↑?		↑?
rs17735961 (CCL11)						?
rs17809012 (CCL11)						?
rs5930 (LDLR)						?
rs11669576 (LDLR)						?
rs11079727 (MAPT) – based on eQTL results		↑				↑
rs1467967 (MAPT)	↓	↓				↓
rs2292305 (THBS1)						?
rs1061624 (TNFR2)						?
						
Overall	↓	↓	↓	↓	↓	↓

Regarding the initial phase of NSPC self-renewal, all variants that could influence this stage, which are rs1800630 and rs1800629 (TNFα), rs11666254 (FPR2), and rs1467967 (MAPT), seem to impair the survival of the cells and thereby might lead to a depletion of the progenitor cell pool. This effect would be mediated by elevated TNFα production and increased tau levels, both of which are implicated chronic inflammatory conditions. Rs522807 (TNFR2) might also participate in the regulation of this stage but since there is a discrepancy between the literature and the eQTL results, it remains unclear if that influence would be protective or further exacerbate the reduction of NSPC self-renewal. Regardless, the HPC0A07/03C cells’ genotype seems to impair NSPC survival by promoting the expression of a pro-inflammatory cytokine.

Proliferation of NSPCs may be inhibited by rs1800630 and rs1800629 (TNFα), rs222747 (TRPV1), rs11666254 (FPR2), and rs1467967 (MAPT) whereby the neurotoxic effect arises due to excessive TNFα, TRPV1, tau or glutamate levels, respectively. Some SNPs could counteract this by reducing IDO activity (rs10108662, IDO1), tau production (rs11079727, MAPT) and by promoting COX2 overexpression (rs5275 and rs689470, PTGS2) but it remains to be tested whether these processes are sufficient to protect the cells from the reductive effects on NSPC proliferation as the latter quantitatively outweigh the others.

Only 1 variant, rs11666254 (FPR2), was found to play a role for NSPC migration whereby its decreasing impact on FPR2 expression potentially disturbs the migration process.

Neurogenesis also seems to be inhibited during the differentiation phase in that rs1800630 and rs1800629 (TNFα) as well as rs11666254 (FPR2) potentially drive the NSPCs towards astrogliogenesis, rather than towards a neuronal fate, by upregulating TNFα expression. This suggests that under chronic inflammation, where neurogenesis is already compromised, the HPC0A07/03C cells generate even less neurons due to their genotype.

When it comes to the survival of newborn neurons, rs689470 and rs5275 (PTGS2), rs1800630 and rs1800629 (TNFα) as well as rs222747 (TRPV1) might exert a negative impact by increasing TNFα, COX2 and TRPV1 expression, respectively. Since rs11666254 (FPR2) has been related to both decreased FPR2 and increased TNFα levels, this SNP could have either a protective or toxic effect or these mechanisms could balance each other out. Either way, the HPC0A07/03C cells’ genotype seems to reduce the capacity of newborn neurons to survive.

Considering the overall impact of each individual variant, when their effects on different stages of hippocampal neurogenesis are summarized and those with controversial results are disregarded, 6 of the SNPs identified in the HPC0A07/03C cell line's genotype according to the criteria applied in this study reduce neurogenesis, while only 2 seem to have a protective influence. This indicates that the cells are more prone to inflammation-mediated toxicity and the resulting impairment of hippocampal neurogenesis. This is largely due to SNPs that are associated with TNFα overexpression.

Before discussing the implications of these findings further, it should be mentioned that the proposed impacts are tentative for two main reasons. For one, findings in previous experiments are contradictory in some cases. For instance, while there is some evidence that rs5275 (PTGS2) leads to COX2 overexpression (Moore et al., 2012), another study did not find this effect (Piranda et al., 2017). A further example is rs11666254 (FPR2) which has been linked to both neuroprotective and neurotoxic effects in that it downregulates FPR2 and simultaneously increases TNFα expression (Zhang et al., 2017a). Just from looking at the literature, it does not become apparent how these two effects interact and if one outweighs the other. The same applies to variants that are implicated in diverse molecular pathways, such as rs222747 (TRPV1) (Mori et al., 2012; Deering-Rice et al., 2016), which seem to affect hippocampal neurogenesis through various mechanisms and the ultimate influence remains ambiguous.

Additionally, the information extracted from the eQTL database is only partially coherent with the literature findings. This becomes most evident for rs522807 (TNFR2), which Fairfax et al. (2011) found to be associated with increased TNFR2 levels, whereas the eQTL database showed reduced TNFR gene expression. This is possibly due to the reported experiment not being focused on hippocampal neurons but on peripheral blood cells, suggesting that the effect of rs522807 on TNFR2 gene expression is cell specific. Another example for the discrepancy between the literature and the eQTL results is the MAPT gene within which one SNP, rs1467967, was found to be associated with elevated tau levels in a previous investigation, whereas another SNP, rs11079727, decreases MAPT gene expression in the hippocampus according to the eQTL database. This example shows that it might be possible for two variants to cause contradictory effects even though they are located on the same gene. In this case, it would be interesting to determine the overall impact of the gene to see whether the effects of the SNPs on gene expression balance each other out overall or whether the effect of one SNP dominates. Again, the different results could be attributed to the cited study (Babić Leko et al., 2018) measuring CSF levels and the eQTL analysis being focused on hippocampal tissue. Finally, the fact that the eQTL calculator tool indicated that only 3 of the selected variants significantly change gene expression levels in the hippocampus, even though the literature suggested that a total of 8 polymorphisms modulate mRNA expression, also calls for a need to validate the change of gene expression directly in the laboratory. This aspect needs to be addressed in further studies.

### 7.2 Implications for the HPC0A07/03C cell line and individuals with similar genetic profiles

On the cellular level, the overall reduction of hippocampal neurogenesis that might be caused by variation in inflammation-related genes in the HPC0A07/03C cells’ genotype means that impairments of the neurogenic process under chronic inflammatory environments might be exacerbated. Consequently, when the cells are used to model neurogenesis in the context of inflammatory challenges or diseases associated with sustained inflammation, their response will be influenced by their genetic composition in the inflammatory network and cannot be generalized onto other NSC lines as cell models with a more protective genotype will probably display a less profound reduction of neurogenesis under the same conditions.

On the behavioural level, these genetically determined changes to the neurogenic process under inflammation might be linked to cognitive deficits and mood disorders. Chronic immune system activation has been associated with impaired performance particularly in hippocampus-dependent tasks which indicates that the reduction of neurogenesis caused by inflammation potentially contributes to impairments in cognition (Kohman and Rhodes, 2013). For instance, patients with Diabetes mellitus or obesity, conditions which lead to chronic peripheral inflammation and have resulted in reduced neurogenesis in animal models, often display deficits in learning and memory as well as depression-like symptoms and show an elevated risk of developing dementia or cognitive impairment (Chesnokova et al., 2016). Individuals with the same or similar genetic alterations as observed in the HPC0A07/03C cell line might be more susceptible to these adverse effects on cognitive and emotional functions since their NSCs are more prone to damage caused by inflammation. These individuals might show more pronounced impairments in hippocampus-dependent tasks such as pattern separation and spatial contextual navigation under chronic inflammatory conditions compared to other subjects with a more protective genotype. They might also be at higher risk for neuropsychiatric disorders like depression and dementia.

### 7.3 Future directions

This report only constitutes a hypothesis on a potential influence of the HPC0A07/03C cell line's genotype on neurogenesis. Whether this hypothesis holds true in reality remains to be evaluated in future studies. The first step here should be to validate the aforementioned information. This includes a confirmation of the genotyping array data of the HPC0A07/03C cell line which should involve a direct genotyping method like Sanger sequencing, for instance. Similarly, further studies should validate whether gene expression levels would change in the hippocampus due to the respective SNPs as suggested in either previous literature or, more importantly, in the eQTL database. Since the studies mentioned here did not investigate hippocampal tissue and the sources of the eQTL database application used here are not clear in detail, this step is necessary to confirm the assumptions made in this project. Subsequently, the HPC0A07/03C cells should be exposed to pro-inflammatory stimuli, in particular TNFα as the expression of this cytokine seems to be modulated by various SNPs identified in the cells' genotype. Other inflammatory stimuli should also be tested to determine the stimulus-specificity of the cells' responses. This may include co-culture with LPS-activated microglia and/or the treatment of HPC0A07/03C with their conditioned medium. In addition, other molecules associated with chronic inflammatory conditions could be included in the cell culture, for instance tau-protein which is involved in the pathophysiology of many neurodegenerative disorders. It is also important to look at different stages of neurogenesis separately because they might be affected in different ways. The findings of these experiments should be compared to other NSC lines including primary hippocampal cells and cells derived from other species to gain a more comprehensive understanding of how different genotypes can affect the neurogenic process. Finally, it would be interesting to determine whether the genetic alterations to cellular functions cause changes in behaviour. To this end, studies using genetically modified animal models and involving hippocampus-dependent tasks like the Morris Water Maze and tests for depressive-like symptoms, for example the forced swim test, could be performed to observe whether animals with a pro-inflammatory genotype perform at a lower level than others.

The finding that the HPC0A07/03C cell line's genotype potentially increases the negative impact of chronic inflammation on all stages of neurogenesis emphasises that it is necessary to consider possible confounding factors that could decrease the generalizability of in vitro experiments. Cell culture models are supposed to be representative for a certain sub-population but since they are derived from individuals with a unique genetic background, their genotype will inevitably influence the outcome of any experiment. Therefore, it is essential to be aware of even subtle genetic variations in the cell line's genome that could alter the response to certain stimuli so that the interaction between experimental conditions and the genotype can be evaluated. This way, results are not overinterpreted and it becomes clear that they only apply to subjects with a similar genotype, at least when it comes to genes that are involved in the processes tested in the respective study.

For these reasons, future studies using cell models should characterize the genome of the chosen cell line, ideally in combination with collecting information on gene expression and gene interactions and include this knowledge in the interpretation of their results. Accordingly, previously published studies should be re-evaluated when new genetic information becomes available. This would limit false conclusions and contribute to the reproducibility of research as unwanted confounding factors related to individual genetics can be taken into account.

## 8 Limitations

Despite the valuable conclusions of this study, it has some limitations that should be considered when interpreting the results of this project.

Firstly, due to the focus on chronic and neuroinflammatory response, only a subset of the genes that are involved in regulating inflammation were analysed here. Consequently, it is possible that some genetic variations that play an important part in modulating the response of HPCs to inflammatory stimuli are not included and thus, the actual phenotype of the HPC0A07/03C cells could differ from what is proposed in this context.

Secondly, this report only constitutes a hypothesis on how the HPC0A07/03C cell line's genotype might alter their inflammatory response since this study was literature-based and was not validated by performing respective experiments with the cell line of interest. A critical caveat related to the chosen approach is that only a fraction of the variants found in the HPC0A07/03C cell line's genotype yielded results in the literature search. It is uncertain whether that is because the respective SNPs were not studied before or because they do not affect the phenotype. Thus, it is likely that more SNPs contribute to the regulation of inflammation in the cell line although they are not mentioned here. Another limitation is that none of the selected SNPs were previously investigated in neurons or NSCs. Instead, the cited studies which show genetic or cellular effects of those variants were performed on other cell types. As the effect of genetic variations can be cell specific, there is a possibility that those findings do not apply to neurons or HPCs in particular. Therefore, it is necessary to test the hypothesis which results from consulting the existing literature in future studies to elucidate the cells' actual behaviour before any further conclusions are drawn. One possibility to get a clearer picture of the phenotype of the cells would be to create a polygenic risk score for the HPC0A07/03C cell line. This method can be used to predict the genetic predisposition for common diseases, like Alzheimer's Disease, by calculating a weighted sum of the number of risk alleles in an individual's genotype, whereby the locations and the contributions of risk alleles are based on results of genome-wide association studies (Lambert et al., 2019; Torkamani et al., 2018). Since this approach aggregates the effects of multiple variants into an overall score, rather than focusing on individual SNPs on their own, it could provide a more refined estimate of the disease risk of the cells.

## 9 Conclusion

This study has provided preliminary evidence that genetic variation on genes associated with inflammation within a specific NSC line, HPC0A07/03C, might aggravate the cells’ response to chronic inflammation by reducing NSPC self-renewal, proliferation, migration, their differentiation into neurons and the survival of newborn neurons. Since these cells are used in in vitro experiments on neurogenesis, which is known to be affected by inflammatory conditions, these results are relevant for determining the implications of those experiments.

Although these findings still need to be confirmed by experimental studies, they highlight the need to characterize the genotype of in vitro models used to investigate cellular mechanisms in healthy or pathological conditions in order to avoid ambiguity and overinterpretation of the results and to improve reproducibility. In the future, information on genes that are relevant to the study at hand should be collected and considered during the interpretation of any observations, regardless of whether an established cell line or primary or stem cells derived from an individual are used. Investigators should carefully assess the utility of the of the chosen cell line as a model for the process of interest and keep in mind that results may only generalize on subjects with a certain genotype.

## Funding

We gratefully acknowledge capital equipment funding from the Maudsley Charity (Grant Ref. 980) and Guy’s and St Thomas’s Charity (Grant Ref. STR130505). The cellular work was funded in the Thuret lab with a grant from the Medical Research Council UK (MR/S00484X/1).

## Declaration of competing interest

This study presents independent research supported by the NIHR BioResource Centre Maudsley, National Institute for Health Research Maudsley Biomedical Research Centre (BRC) at South London and Maudsley NHS Foundation Trust and Institute of Psychiatry, Psychology and Neuroscience (IoPPN), King's College London. The views expressed are those of the author(s) and not necessarily those of the NHS, NIHR, Department of Health or King's College London. The authors declare no competing interests.
