# Supplementary material for: The effects of genotype on inflammatory response in hippocampal progenitor cells: A computational approach
Source: Brain Behav Immun Health. 2021 Jun 15;15:100286. doi: 10.1016/j.bbih.2021.100286 (PMC8261829; doi:10.1016/j.bbih.2021.100286)
Supplement: Multimedia component 1 [file mmc1.docx]

# **Supplementary Materials (Lee, Metz et al.)**

# **R Script for variant extraction**

## **Loading necessary packages**

#if necessary, install and load packages (stringr and dplyr)

if (!suppressWarnings(require(stringr))) install.packages("stringr")

if (!suppressWarnings(require(dplyr))) install.packages("dplyr")

library(stringr)

library(dplyr)

## **Filtering of VEP results**

#load VEP results dataset (includes only variants from the genotyping array data that match an existing variant and have related publications on PubMed) in workspace

VEP <- read.delim("~/RX5q3EH7yJFHOk9z.Existing_variation_and_PUBMED.vcf", header=FALSE, comment.char="#", stringsAsFactors=TRUE)

#delete empty columns and missing genotypes, rename columns

VEP[6:7] <- list(NULL)

VEP[7] <- list(NULL)

names(VEP) <- c("CHROM","POS","ID","REF","ALT","INFO","GT")

VEP <- subset(VEP, `GT`!= "./.")

#split the "INFO" column into its sub-parts and rename the columns

VEP <- cbind(VEP,str_split_fixed(VEP$INFO, "[|]", 38))

names(VEP) <- c("CHROM","POS","ID","REF","ALT","INFO","GT","Allele","Consequence","IMPACT","SYMBOL",

"Gene","Feature_type","Feature","BIOTYPE","EXON","INTRON","HGVSc","HGVSp","cDNA_position","CDS_position","Protein_position","Amino_acids","Codons","Existing_variation","DISTANCE","STRAND","FLAGS","SYMBOL_SOURCE","HGNC_ID","MANE","TSL","APPRIS","SIFT","PolyPhen","AF","CLIN_SIG","SOMATIC","PHENO","PUBMED","MOTIF_NAME","MOTIF_POS","HIGH_INF_POS","MOTIF_SCORE_CHANGE","other")

#remove empty column

VEP[6] <- list(NULL)

#remove duplicated variants based on their existing variant ID

VEP <- VEP[!duplicated(VEP$Existing_variation), ]

## **Extraction of variants based on AmiGO gene lists**

### **Gene ontology term “chronic inflammatory response”**

#extract variants on genes annotated with "chronic inflammatory response" on AmiGO

#load AmiGO gene list onto workspace, create vector from "gene label" column and extract that from VEP dataset

GOchronicinflammatoryresponse <- read.delim("~/GO_chronicinflammatoryresponse.txt", header=FALSE, stringsAsFactors=TRUE)

GOchronicinflammatoryresponse <- as.vector(GOchronicinflammatoryresponse$V1)

VEPchronicinflammatoryresponse <- filter(VEP, SYMBOL %in% GOchronicinflammatoryresponse)

### **Gene ontology term “neuroinflammatory response”**

#extract variants on genes annotated with "neuroinflammatory response" on AmiGO

GOneuroinflammatoryresponse <- read.delim("~/GO_neuroinflammatoryresponse.txt", header=FALSE, stringsAsFactors=TRUE)

GOneuroinflammatoryresponse <-as.vector(GOneuroinflammatoryresponse$V1)

VEPneuroinflammatoryresponse <- filter(VEP, SYMBOL %in% GOneuroinflammatoryresponse)

### **Gene ontology term “neurogenesis”**

#determine which of the previously extracted variants are also related to neurogenesis

#extract variants on genes annotated with "neurogenesis" on AmiGO

GOneurogenesis <- read.delim("~/GO_neurogenesis.txt", header=FALSE, stringsAsFactors=TRUE)

GOneurogenesis <- as.vector(GOneurogenesis$V1)

VEPneurogenesis <- filter(VEP, SYMBOL %in% GOneurogenesis)

#combine neurogenesis and chronic inflammatory response/neuroinflammatory response lists

VEPchronicinflammatoryresponseANDneurogenesis <- filter(VEPneurogenesis, SYMBOL %in% GOchronicinflammatoryresponse)

VEPneuroinflammatoryresponseANDneurogenesis <- filter(VEPneurogenesis, SYMBOL %in% GOneuroinflammatoryresponse)

# **Lists of genes retrieved from AmiGO**

**Table 2.1**. List of genes annotated to the gene ontology term "chronic inflammatory response" as retrieved from the AmiGO application

| Gene label | Gene name | Gene label | Gene name |
| --- | --- | --- | --- |
| ADORA2B | Adenosine receptor A2b | **IL10** | Interleukin-10 |
| AHCY | Adenosylhomocysteinase | **LTA** | Lymphotoxin-alpha |
| CCL5 | C-C motif chemokine 5 | **PTGES** | Prostaglandin E synthase |
| CCL11 | Eotaxin | **S100A8** | Protein S100-A8 |
| CXCL13 | C-X-C motif chemokine 13 | **THBS1** | Thrombospondin-1 |
| CYP19A1 | Aromatase | **TNF** | Tumor necrosis factor |
| FOXP3 | Forkhead box protein P3 | **TNFAIP3** | Tumor necrosis factor alpha-induced protein 3 |
| GJA1 | Gap junction alpha-1 protein | **UNC13D** | Protein unc-13 homolog D |
| IDO1 | Indoleamine 2,3-dioxygenase 1 | **VNN1** | Pantetheinase |
| IL4 | Interleukin-4 |  |  |

**Table 2.2**. List of genes annotated to the gene ontology term "neuroinflammatory response" as retrieved from AmiGO

| Gene label | Gene name | Gene label | Gene name |
| --- | --- | --- | --- |
| ADCY1 | Adenylate cyclase type 1 | **ITGB1** | Integrin beta-1 |
| ADCY8 | Adenylate cyclase type 8 | **ITGB2** | Integrin beta-2 |
| ADORA2A | Adenosine receptor A2a | **JAK2** | Tyrosine-protein kinase JAK2 |
| AGER | Advanced glycosylation end product-specific receptor | **JUN** | Transcription factor AP-1 |
| AIF1 | Allograft inflammatory factor 1 | **LDLR** | Low-density lipoprotein receptor |
| APP | Amyloid-beta precursor protein | **LRP1** | Prolow-density lipoprotein receptor-related protein 1 |
| ATM | Serine-protein kinase ATM | **LRRK2** | Leucine-rich repeat serine/threonine-protein kinase 2 |
| AZU1 | Azurocidin | **MAPT** | Microtubule-associated protein tau |
| BACE2 | Beta-secretase 2 | **MMP3** | Stromelysin-1 |
| C1QA | Complement C1q subcomponent subunit A | **MMP8** | Neutrophil collagenase |
| C5AR1 | C5a anaphylatoxin chemotactic receptor 1 | **MMP9** | Matrix metalloproteinase-9 |
| CCL3 | C-C motif chemokine 3 | **NAMPT** | Nicotinamide phosphoribosyltransferase |
| CD200 | OX-2 membrane glycoprotein | **NR1D1** | Nuclear receptor subfamily 1 group D member 1 |
| CD200R1 | Cell surface glycoprotein CD200 receptor 1 | **NUPR1** | Nuclear protein 1 |
| CD200R1L | Cell surface glycoprotein CD200 receptor 2 | **PSEN1** | Presenilin-1 |
| CLU | Clusterin | **PTGS2** | Prostaglandin G/H synthase 2 |
| CNTF | Ciliary neurotrophic factor | **PTPRC** | Receptor-type tyrosine-protein phosphatase C |
| CST7 | Cystatin-F | **SMO** | Smoothened homolog |
| CTSC | Dipeptidyl peptidase 1 | **SNCA** | Alpha-synuclein |
| CX3CL1 | Fractalkine | **SPHK1** | Sphingosine kinase 1 |
| DAGLA | Sn1-specific diacylglycerol lipase alpha | **STAP1** | Signal-transducing adaptor protein 1 |
| EGFR | Epidermal growth factor receptor | **SYT11** | Synaptotagmin-11 |
| FPR2 | N-formyl peptide receptor 2 | **TAFA3** | Chemokine-like protein TAFA-3 |
| GRN | Progranulin | **TLR2** | Toll-like receptor 2 |
| IFNG | Interferon gamma | **TLR3** | Toll-like receptor 3 |
| IFNGR1 | Interferon gamma receptor 1 | **TLR6** | Toll-like receptor 6 |
| IGF1 | Insulin-like growth factor I | **TLR7** | Toll-like receptor 7 |
| IL13 | Interleukin-13 | **TLR8** | Toll-like receptor 8 |
| IL18 | Interleukin-18 | **TNF** | Tumor necrosis factor |
| IL1B | Interleukin-1 beta | **TNFRSF1B** | Tumor necrosis factor receptor superfamily member 1B |
| IL33 | Interleukin-33 | **TREM2** | Triggering receptor expressed on myeloid cells 2 |
| IL4 | Interleukin-4 | **TRPV1** | Transient receptor potential cation channel subfamily V member 1 |
| IL6 | Interleukin-6 | **TTBK1** | Tau-tubulin kinase 1 |
| ITGAM | Integrin alpha-M | **TYROBP** | TYRO protein tyrosine kinase-binding protein |

**Table 2.3**. List of genes annotated to both the gene ontology term "neurogenesis" and either “chronic inflammatory response” or “neuroinflammatory response”, as retrieved from AmiGO

| Gene label | Gene name | Gene label | Gene name |
| --- | --- | --- | --- |
| ADCY1 | Adenylate cyclase type 1 | **IL1B** | Interleukin-1 beta |
| ADORA2A | Adenosine receptor A2a | **IL33** | Interleukin-33 |
| AGER | Advanced glycosylation end product-specific receptor | **IL6** | Interleukin-6 |
| APP | Amyloid-beta precursor protein | **ITGB1** | Integrin beta-1 |
| AZU1 | Azurocidin | **JAK2** | Tyrosine-protein kinase JAK2 |
| BACE2 | Beta-secretase 2 | **JUN** | Transcription factor AP-1 |
| C1QA | Complement C1q subcomponent subunit A | **LDLR** | Low-density lipoprotein receptor |
| C5AR1 | C5a anaphylatoxin chemotactic receptor 1 | **LRP1** | Prolow-density lipoprotein receptor-related protein 1 |
| CCL11 | Eotaxin | **LRRK2** | Leucine-rich repeat serine/threonine-protein kinase 2 |
| CCL3 | C-C motif chemokine 3 | **LTA** | Lymphotoxin-alpha |
| CLU | Clusterin | **MAPT** | Microtubule-associated protein tau |
| CNTF | Ciliary neurotrophic factor | **NR1D1** | Nuclear receptor subfamily 1 group D member 1 |
| CX3CL1 | Fractalkine | **PSEN1** | Presenilin-1 |
| EGFR | Epidermal growth factor receptor | **S100A8** | Protein S100-A8 |
| FPR2 | N-formyl peptide receptor 2 | **SMO** | Smoothened homolog |
| GJA1 | Gap junction alpha-1 protein | **TAFA3** | Chemokine-like protein TAFA-3 |
| GRN | Progranulin | **TLR2** | Toll-like receptor 2 |
| IFNG | Interferon gamma | **TNF** | Tumor necrosis factor |
| IFNGR1 | Interferon gamma receptor 1 | **TNFRSF1B** | Tumor necrosis factor receptor superfamily member 1B |

# **Lists of variants extracted from the genotyping array data**

**Table 3.1**. List of variants in the HPC0A07/03C cells' genotype that are located on genes annotated to "chronic inflammatory response" (in alphabetical order of the gene label). Genes which are also annotated with the GOterm “neurogenesis” are marked in bold. Key for the genotype information: 0/0: homozygous for the reference allele; 1/1: homozygous for the alternative allele; 0/1: heterozygous.

| Variant ID | Chromo-some | Position | Gene label | Gene ID | Reference allele | Alternative allele | Genotype in HPC0A07/03C cells |
| --- | --- | --- | --- | --- | --- | --- | --- |
| rs17735961 | 17 | 32612340 | **CCL11** | ENSG00000172156 | C | A | 0/0 |
| rs17809012 | 17 | 32612444 | **CCL11** | ENSG00000172156 | A | G | 0/0 |
| rs714910 | 17 | 32617265 | **CCL11** | ENSG00000172156 | A | C | 1/1 |
| rs1860181 | 17 | 32617587 | **CCL11** | ENSG00000172156 | A | G | 0/0 |
| rs10046 | 15 | 51502986 | CYP19A1 | ENSG00000137869 | G | A | 0/1 |
| rs2304461 | 15 | 51504556 | CYP19A1 | ENSG00000137869 | G | A | 0/0 |
| rs17601241 | 15 | 51507874 | CYP19A1 | ENSG00000137869 | G | A | 0/1 |
| rs700519 | 15 | 51507968 | CYP19A1 | ENSG00000137869 | G | A | 0/0 |
| rs2899472 | 15 | 51516055 | CYP19A1 | ENSG00000137869 | C | A | 0/0 |
| rs9944225 | 15 | 51528080 | CYP19A1 | ENSG00000137869 | C | A | 0/1 |
| rs12591359 | 15 | 51539368 | CYP19A1 | ENSG00000137869 | G | A | 0/1 |
| rs2305707 | 15 | 51569410 | CYP19A1 | ENSG00000137869 | A | G | 0/1 |
| rs10519301 | 15 | 51599537 | CYP19A1 | ENSG00000137869 | G | A | 0/1 |
| rs10519302 | 15 | 51599683 | CYP19A1 | ENSG00000137869 | A | G | 0/1 |
| rs4774585 | 15 | 51616480 | CYP19A1 | ENSG00000137869 | G | A | 0/1 |
| rs199809018 | X | 49103192 | FOXP3 | ENSG00000049768 | G | A | 0/0 |
| rs140735182 | X | 49104009 | FOXP3 | ENSG00000049768 | G | A | 0/0 |
| rs144632022 | X | 49104943 | FOXP3 | ENSG00000049768 | G | A | 0/0 |
| rs147222955 | X | 49105970 | FOXP3 | ENSG00000049768 | G | A | 0/0 |
| rs2232367 | X | 49113312 | FOXP3 | ENSG00000049768 | G | A | 0/0 |
| rs200554980 | X | 49113998 | FOXP3 | ENSG00000049768 | G | A | 0/0 |
| rs3761549 | X | 49117345 | FOXP3 | ENSG00000049768 | G | A | 0/1 |
| rs35059413 | 8 | 39771451 | IDO1 | ENSG00000131203 | G | A | 0/0 |
| rs35099072 | 8 | 39775653 | IDO1 | ENSG00000131203 | G | A | 0/0 |
| rs12545877 | 8 | 39776344 | IDO1 | ENSG00000131203 | G | A | 0/0 |
| rs10108662 | 8 | 39779989 | IDO1 | ENSG00000131203 | C | A | 0/1 |
| rs3739319 | 8 | 39785321 | IDO1 | ENSG00000131203 | G | A | 0/1 |
| rs3024505 | 1 | 206939904 | IL10 | ENSG00000136634 | G | A | 0/0 |
| rs3024496 | 1 | 206941864 | IL10 | ENSG00000136634 | A | G | 0/0 |
| rs3024493 | 1 | 206943968 | IL10 | ENSG00000136634 | C | A | 0/0 |
| rs56279116 | 5 | 132010172 | IL4 | ENSG00000113520 | G | A | 0/0 |
| rs2243268 | 5 | 132013963 | IL4 | ENSG00000113520 | A | C | 0/1 |
| rs2243270 | 5 | 132014109 | IL4 | ENSG00000113520 | A | G | 0/1 |
| rs2009658 | 6 | 31538244 | **LTA** | ENSG00000226979 | C | G | 0/0 |
| rs915654 | 6 | 31538497 | **LTA** | ENSG00000226979 | T | A | 0/0 |
| rs1800630 | 6 | 31542476 | **LTA** | ENSG00000226979 | C | A | 0/0 |
| rs1800629 | 6 | 31543031 | **LTA** | ENSG00000226979 | G | A | 0/1 |
| rs3093662 | 6 | 31544189 | **LTA** | ENSG00000226979 | A | G | 0/0 |
| rs3093671 | 6 | 31546980 | **LTA** | ENSG00000226979 | G | A | 0/0 |
| rs2302821 | 9 | 132501881 | PTGES | ENSG00000148344 | A | C | 1/1 |
| rs11792431 | 9 | 132515247 | PTGES | ENSG00000148344 | G | A | 0/0 |
| rs2292305 | 15 | 39880822 | THBS1 | ENSG00000137801 | A | G | 0/1 |
| rs143790069 | 15 | 39883738 | THBS1 | ENSG00000137801 | G | A | 0/0 |
| rs3743125 | 15 | 39887848 | THBS1 | ENSG00000137801 | G | A | 0/1 |
| rs5029933 | 6 | 138192062 | TNFAIP3 | ENSG00000118503 | A | G | 0/0 |
| rs719150 | 6 | 138192761 | TNFAIP3 | ENSG00000118503 | A | G | 0/0 |
| rs661561 | 6 | 138197331 | TNFAIP3 | ENSG00000118503 | A | C | 0/1 |
| rs200840068 | 6 | 138198246 | TNFAIP3 | ENSG00000118503 | G | A | 0/0 |
| rs150355046 | 6 | 138202314 | TNFAIP3 | ENSG00000118503 | G | A | 0/0 |
| rs118049905 | 17 | 73826167 | UNC13D | ENSG00000092929 | G | A | 0/0 |
| rs35037984 | 17 | 73826491 | UNC13D | ENSG00000092929 | G | A | 0/0 |
| rs2290770 | 17 | 73831016 | UNC13D | ENSG00000092929 | G | A | 0/0 |
| rs143305366 | 17 | 73831848 | UNC13D | ENSG00000092929 | C | A | 0/0 |
| rs75366116 | 17 | 73831953 | UNC13D | ENSG00000092929 | G | A | 0/0 |
| rs121434352 | 17 | 73836398 | UNC13D | ENSG00000092929 | G | A | 0/0 |

**Table 3.2**. List of variants in the HPC0A07/03C cells' genotype that are located on genes annotated to "neuroinflammatory response" (in alphabetical order of the gene label). Genes which are also annotated with the GOterm “neurogenesis” are marked in bold. Key for the genotype information: 0/0: homozygous for the reference allele; 1/1: homozygous for the alternative allele; 0/1: heterozygous.

| Variant ID | Chromo-some | Position | Gene label | Gene ID | Reference allele | Alternative allele | Genotype in HPC0A07/03C cells |
| --- | --- | --- | --- | --- | --- | --- | --- |
| rs1042009 | 7 | 45703971 | **ADCY1** | ENSG00000164742 | G | A | 0/0 |
| rs263238 | 8 | 131812033 | ADCY8 | ENSG00000155897 | G | A | 0/0 |
| rs1435446 | 8 | 131826621 | ADCY8 | ENSG00000155897 | G | A | 0/0 |
| rs10505566 | 8 | 131879068 | ADCY8 | ENSG00000155897 | A | C | 0/0 |
| rs965814 | 8 | 131894883 | ADCY8 | ENSG00000155897 | G | A | 1/1 |
| rs7016933 | 8 | 131903134 | ADCY8 | ENSG00000155897 | A | G | 0/0 |
| rs12547243 | 8 | 131921956 | ADCY8 | ENSG00000155897 | A | G | 0/0 |
| rs6986982 | 8 | 131987375 | ADCY8 | ENSG00000155897 | G | A | 0/1 |
| rs2228950 | 8 | 132002770 | ADCY8 | ENSG00000155897 | G | A | 0/1 |
| rs885381 | 8 | 132023602 | ADCY8 | ENSG00000155897 | G | A | 0/0 |
| rs3132451 | 6 | 31582025 | AIF1 | ENSG00000204472 | G | C | 0/1 |
| rs28732150 | 6 | 31583224 | AIF1 | ENSG00000204472 | G | A | 0/0 |
| rs2736182 | 6 | 31583312 | AIF1 | ENSG00000204472 | G | A | 0/0 |
| rs373521 | 21 | 27257660 | **APP** | ENSG00000142192 | C | A | 0/0 |
| rs2829973 | 21 | 27260456 | **APP** | ENSG00000142192 | A | G | 1/1 |
| rs214488 | 21 | 27287924 | **APP** | ENSG00000142192 | G | A | 0/0 |
| rs2830012 | 21 | 27397607 | **APP** | ENSG00000142192 | A | G | 0/1 |
| rs1041420 | 21 | 27443650 | **APP** | ENSG00000142192 | G | A | 0/0 |
| rs9984764 | 21 | 27497497 | **APP** | ENSG00000142192 | G | A | 0/0 |
| rs12482753 | 21 | 27513837 | **APP** | ENSG00000142192 | A | G | 0/0 |
| rs1800054 | 11 | 108098576 | ATM | ENSG00000149311 | C | G | 0/0 |
| rs142358238 | 11 | 108100039 | ATM | ENSG00000149311 | G | A | 0/0 |
| rs146382972 | 11 | 108106399 | ATM | ENSG00000149311 | G | A | 0/0 |
| rs2235000 | 11 | 108121733 | ATM | ENSG00000149311 | G | A | 0/0 |
| rs3218695 | 11 | 108129778 | ATM | ENSG00000149311 | C | A | 0/0 |
| rs11212570 | 11 | 108130712 | ATM | ENSG00000149311 | G | A | 0/0 |
| rs149711770 | 11 | 108155132 | ATM | ENSG00000149311 | G | A | 0/0 |
| rs145667735 | 11 | 108160467 | ATM | ENSG00000149311 | G | A | 0/0 |
| rs55870064 | 11 | 108168053 | ATM | ENSG00000149311 | A | G | 0/0 |
| rs1801516 | 11 | 108175462 | ATM | ENSG00000149311 | G | A | 0/0 |
| rs147187700 | 11 | 108180945 | ATM | ENSG00000149311 | G | C | 0/0 |
| rs11212587 | 11 | 108186610 | ATM | ENSG00000149311 | G | A | 0/0 |
| rs139770721 | 11 | 108186638 | ATM | ENSG00000149311 | G | A | 0/0 |
| rs1800060 | 11 | 108188136 | ATM | ENSG00000149311 | G | A | 0/0 |
| rs56815840 | 11 | 108190770 | ATM | ENSG00000149311 | G | C | 0/0 |
| rs28626600 | 19 | 830820 | **AZU1** | ENSG00000172232 | G | A | 0/0 |
| rs6517656 | 21 | 42583738 | **BACE2** | ENSG00000182240 | G | A | 0/0 |
| rs2837990 | 21 | 42620149 | **BACE2** | ENSG00000182240 | G | A | 0/0 |
| rs2837994 | 21 | 42624124 | **BACE2** | ENSG00000182240 | G | A | 0/0 |
| rs11702001 | 21 | 42627969 | **BACE2** | ENSG00000182240 | G | A | 0/0 |
| rs8133778 | 21 | 42642038 | **BACE2** | ENSG00000182240 | A | G | 1/1 |
| rs1072869 | 21 | 42657548 | **BACE2** | ENSG00000182240 | A | C | 0/1 |
| rs4577202 | 19 | 47815922 | **C5AR1** | ENSG00000197405 | A | G | 0/1 |
| rs9972960 | 17 | 34420079 | **CCL3** | ENSG00000006075 | G | A | 0/0 |
| rs1131199 | 3 | 112059768 | CD200 | ENSG00000091972 | C | G | 0/0 |
| rs2272022 | 3 | 112063850 | CD200 | ENSG00000091972 | C | A | 0/0 |
| rs1050572 | 3 | 112066562 | CD200 | ENSG00000091972 | G | A | 1/1 |
| rs7640752 | 3 | 112640446 | CD200R1 | ENSG00000163606 | G | A | 0/0 |
| rs9331947 | 8 | 27454877 | **CLU** | ENSG00000120885 | A | G | 0/1 |
| rs9314349 | 8 | 27474202 | **CLU** | ENSG00000120885 | A | G | 0/1 |
| rs17594 | 11 | 88027393 | CTSC | ENSG00000109861 | A | C | 0/0 |
| rs763317 | 7 | 55095197 | **EGFR** | ENSG00000146648 | A | G | 1/1 |
| rs10488137 | 7 | 55112827 | **EGFR** | ENSG00000146648 | G | A | 0/0 |
| rs17172433 | 7 | 55142435 | **EGFR** | ENSG00000146648 | G | A | 0/0 |
| rs10245472 | 7 | 55147478 | **EGFR** | ENSG00000146648 | G | A | 0/0 |
| rs10244108 | 7 | 55152337 | **EGFR** | ENSG00000146648 | G | A | 0/0 |
| rs6954351 | 7 | 55171190 | **EGFR** | ENSG00000146648 | G | A | 0/0 |
| rs7796872 | 7 | 55179844 | **EGFR** | ENSG00000146648 | G | A | 0/1 |
| rs13244925 | 7 | 55192256 | **EGFR** | ENSG00000146648 | A | C | 1/1 |
| rs17172446 | 7 | 55202598 | **EGFR** | ENSG00000146648 | G | A | 0/1 |
| rs17289589 | 7 | 55211050 | **EGFR** | ENSG00000146648 | G | A | 0/0 |
| rs13234622 | 7 | 55211879 | **EGFR** | ENSG00000146648 | A | G | 0/1 |
| rs11506105 | 7 | 55220177 | **EGFR** | ENSG00000146648 | A | G | 0/1 |
| rs2227983 | 7 | 55229255 | **EGFR** | ENSG00000146648 | G | A | 0/1 |
| rs150477666 | 7 | 55229273 | **EGFR** | ENSG00000146648 | G | A | 0/0 |
| rs11976696 | 7 | 55232333 | **EGFR** | ENSG00000146648 | A | G | 0/1 |
| rs845551 | 7 | 55237283 | **EGFR** | ENSG00000146648 | A | G | 1/1 |
| rs10228436 | 7 | 55238268 | **EGFR** | ENSG00000146648 | G | A | 0/1 |
| rs150423237 | 7 | 55240780 | **EGFR** | ENSG00000146648 | G | A | 0/0 |
| rs2017000 | 7 | 55242609 | **EGFR** | ENSG00000146648 | A | G | 0/1 |
| rs9692301 | 7 | 55243754 | **EGFR** | ENSG00000146648 | A | G | 0/1 |
| rs6593210 | 7 | 55254186 | **EGFR** | ENSG00000146648 | G | A | 0/0 |
| rs41321844 | 7 | 55273162 | **EGFR** | ENSG00000146648 | G | A | 0/0 |
| rs1107617 | 7 | 55280268 | **EGFR** | ENSG00000146648 | A | C | 0/0 |
| rs11666254 | 19 | 52263162 | **FPR2** | ENSG00000171049 | A | G | 0/1 |
| rs3785817 | 17 | 42423665 | **GRN** | ENSG00000030582 | A | G | 1/1 |
| rs148531161 | 17 | 42426884 | **GRN** | ENSG00000030582 | G | A | 0/0 |
| rs63750043 | 17 | 42427605 | **GRN** | ENSG00000030582 | C | A | 0/0 |
| rs63750541 | 17 | 42428954 | **GRN** | ENSG00000030582 | G | A | 0/0 |
| rs2234687 | 12 | 68548756 | **IFNG** | ENSG00000111537 | G | A | 0/0 |
| rs2069718 | 12 | 68550162 | **IFNG** | ENSG00000111537 | A | G | 0/1 |
| rs9376268 | 6 | 137532751 | **IFNGR1** | ENSG00000027697 | G | A | 0/0 |
| rs10457655 | 6 | 137539810 | **IFNGR1** | ENSG00000027697 | G | A | 0/1 |
| rs3730204 | 12 | 102795514 | IGF1 | ENSG00000017427 | A | G | 0/1 |
| rs5742632 | 12 | 102856474 | IGF1 | ENSG00000017427 | A | G | 0/1 |
| rs12821878 | 12 | 102867667 | IGF1 | ENSG00000017427 | G | A | 0/0 |
| rs1881457 | 5 | 131992409 | IL13 | ENSG00000169194 | A | C | 0/1 |
| rs848 | 5 | 131996500 | IL13 | ENSG00000169194 | A | C | 0/1 |
| rs2243208 | 5 | 132001151 | IL13 | ENSG00000169194 | A | G | 0/1 |
| rs5744290 | 11 | 112014328 | IL18 | ENSG00000150782 | G | A | 0/0 |
| rs2043055 | 11 | 112031624 | IL18 | ENSG00000150782 | A | G | 0/1 |
| rs3917366 | 2 | 113583880 | **IL1B** | ENSG00000125538 | C | A | 0/0 |
| rs3136558 | 2 | 113591275 | **IL1B** | ENSG00000125538 | A | G | 0/1 |
| rs10435816 | 9 | 6225535 | **IL33** | ENSG00000137033 | A | G | 0/1 |
| rs12551256 | 9 | 6231239 | **IL33** | ENSG00000137033 | A | G | 0/1 |
| rs16924241 | 9 | 6256144 | **IL33** | ENSG00000137033 | C | G | 0/0 |
| rs1048274 | 9 | 6256292 | **IL33** | ENSG00000137033 | G | A | 0/1 |
| rs56279116 | 5 | 132010172 | IL4 | ENSG00000113520 | G | A | 0/0 |
| rs2243268 | 5 | 132013963 | IL4 | ENSG00000113520 | A | C | 0/1 |
| rs2243270 | 5 | 132014109 | IL4 | ENSG00000113520 | A | G | 0/1 |
| rs13306435 | 7 | 22771039 | **IL6** | ENSG00000136244 | T | A | 0/0 |
| rs1143679 | 16 | 31276811 | ITGAM | ENSG00000169896 | G | A | 0/0 |
| rs2359661 | 16 | 31281147 | ITGAM | ENSG00000169896 | A | G | 0/1 |
| rs12928810 | 16 | 31311715 | ITGAM | ENSG00000169896 | G | A | 0/0 |
| rs11150610 | 16 | 31334236 | ITGAM | ENSG00000169896 | C | A | 0/1 |
| rs2153875 | 10 | 33190567 | **ITGB1** | ENSG00000150093 | C | A | 0/1 |
| rs1316757 | 10 | 33197197 | **ITGB1** | ENSG00000150093 | A | C | 0/1 |
| rs3780873 | 10 | 33213680 | **ITGB1** | ENSG00000150093 | G | A | 0/0 |
| rs2298141 | 10 | 33214802 | **ITGB1** | ENSG00000150093 | A | G | 0/0 |
| rs2230394 | 10 | 33217110 | **ITGB1** | ENSG00000150093 | G | A | 0/1 |
| rs5030672 | 21 | 46309312 | ITGB2 | ENSG00000160255 | G | A | 0/0 |
| rs235325 | 21 | 46311264 | ITGB2 | ENSG00000160255 | A | G | 1/1 |
| rs2838729 | 21 | 46319417 | ITGB2 | ENSG00000160255 | A | G | 0/1 |
| rs1041457 | 21 | 46321818 | ITGB2 | ENSG00000160255 | A | G | 0/1 |
| rs3746972 | 21 | 46327835 | ITGB2 | ENSG00000160255 | A | G | 0/1 |
| rs56118985 | 9 | 5044432 | **JAK2** | ENSG00000096968 | G | A | 0/1 |
| rs200018153 | 9 | 5065000 | **JAK2** | ENSG00000096968 | G | A | 0/0 |
| rs10974944 | 9 | 5070831 | **JAK2** | ENSG00000096968 | C | G | 0/1 |
| rs10974947 | 9 | 5072846 | **JAK2** | ENSG00000096968 | G | A | 0/0 |
| rs3780379 | 9 | 5112519 | **JAK2** | ENSG00000096968 | G | A | 0/0 |
| rs41316003 | 9 | 5126343 | **JAK2** | ENSG00000096968 | G | A | 0/0 |
| rs2760496 | 1 | 59242028 | **JUN** | ENSG00000177606 | A | C | 0/1 |
| rs201102461 | 19 | 11215926 | **LDLR** | ENSG00000130164 | G | A | 0/0 |
| rs199774121 | 19 | 11216011 | **LDLR** | ENSG00000130164 | C | A | 0/0 |
| rs200727689 | 19 | 11216084 | **LDLR** | ENSG00000130164 | G | A | 0/0 |
| rs201384282 | 19 | 11216238 | **LDLR** | ENSG00000130164 | G | A | 0/0 |
| rs121908035 | 19 | 11216275 | **LDLR** | ENSG00000130164 | C | A | 0/0 |
| rs148171426 | 19 | 11217256 | **LDLR** | ENSG00000130164 | G | A | 0/0 |
| rs139043155 | 19 | 11217344 | **LDLR** | ENSG00000130164 | T | A | 0/0 |
| rs143992984 | 19 | 11217352 | **LDLR** | ENSG00000130164 | G | A | 0/0 |
| rs2569556 | 19 | 11218965 | **LDLR** | ENSG00000130164 | G | A | 0/1 |
| rs72658860 | 19 | 11221357 | **LDLR** | ENSG00000130164 | G | A | 0/0 |
| rs11669576 | 19 | 11222300 | **LDLR** | ENSG00000130164 | G | A | 0/0 |
| rs142319510 | 19 | 11223991 | **LDLR** | ENSG00000130164 | G | C | 0/0 |
| rs139617694 | 19 | 11224210 | **LDLR** | ENSG00000130164 | G | A | 0/0 |
| rs5930 | 19 | 11224265 | **LDLR** | ENSG00000130164 | A | G | 0/1 |
| rs139624145 | 19 | 11224296 | **LDLR** | ENSG00000130164 | G | A | 0/0 |
| rs141673997 | 19 | 11224398 | **LDLR** | ENSG00000130164 | G | A | 0/0 |
| rs138947766 | 19 | 11227559 | **LDLR** | ENSG00000130164 | G | A | 0/0 |
| rs2738450 | 19 | 11228965 | **LDLR** | ENSG00000130164 | C | A | 0/0 |
| rs145787161 | 19 | 11231199 | **LDLR** | ENSG00000130164 | G | A | 0/0 |
| rs5927 | 19 | 11233941 | **LDLR** | ENSG00000130164 | A | G | 0/1 |
| rs143771219 | 19 | 11238731 | **LDLR** | ENSG00000130164 | G | A | 0/0 |
| rs2569537 | 19 | 11240053 | **LDLR** | ENSG00000130164 | G | A | 0/0 |
| rs5928 | 19 | 11240240 | **LDLR** | ENSG00000130164 | G | A | 0/0 |
| rs3826810 | 19 | 11242133 | **LDLR** | ENSG00000130164 | G | A | 0/0 |
| rs2738465 | 19 | 11242496 | **LDLR** | ENSG00000130164 | G | A | 0/0 |
| rs35890409 | 12 | 57569339 | **LRP1** | ENSG00000123384 | G | A | 0/0 |
| rs1140648 | 12 | 57593101 | **LRP1** | ENSG00000123384 | G | A | 0/1 |
| rs1800159 | 12 | 57593894 | **LRP1** | ENSG00000123384 | A | G | 1/1 |
| rs1800164 | 12 | 57604802 | **LRP1** | ENSG00000123384 | A | G | 0/1 |
| rs11175655 | 12 | 40623727 | **LRRK2** | ENSG00000188906 | G | A | 0/0 |
| rs78501232 | 12 | 40645075 | **LRRK2** | ENSG00000188906 | G | A | 0/0 |
| rs7955902 | 12 | 40645257 | **LRRK2** | ENSG00000188906 | C | A | 0/1 |
| rs1491938 | 12 | 40645630 | **LRRK2** | ENSG00000188906 | G | A | 0/1 |
| rs7135747 | 12 | 40646708 | **LRRK2** | ENSG00000188906 | A | G | 0/0 |
| rs7308720 | 12 | 40657700 | **LRRK2** | ENSG00000188906 | C | G | 0/0 |
| rs10506151 | 12 | 40670998 | **LRRK2** | ENSG00000188906 | C | A | 0/1 |
| rs10784486 | 12 | 40677029 | **LRRK2** | ENSG00000188906 | A | C | 0/1 |
| rs4640000 | 12 | 40699593 | **LRRK2** | ENSG00000188906 | C | G | 0/0 |
| rs72546338 | 12 | 40702283 | **LRRK2** | ENSG00000188906 | G | A | 0/0 |
| rs7133914 | 12 | 40702911 | **LRRK2** | ENSG00000188906 | G | A | 0/0 |
| rs11175964 | 12 | 40702987 | **LRRK2** | ENSG00000188906 | G | A | 0/0 |
| rs111501952 | 12 | 40704263 | **LRRK2** | ENSG00000188906 | G | A | 0/0 |
| rs35507033 | 12 | 40707778 | **LRRK2** | ENSG00000188906 | G | A | 0/0 |
| rs721710 | 12 | 40709068 | **LRRK2** | ENSG00000188906 | T | A | 0/0 |
| rs11564148 | 12 | 40713901 | **LRRK2** | ENSG00000188906 | T | A | 0/1 |
| rs4768230 | 12 | 40715302 | **LRRK2** | ENSG00000188906 | G | A | 0/1 |
| rs11564203 | 12 | 40724581 | **LRRK2** | ENSG00000188906 | G | A | 0/0 |
| rs10735934 | 12 | 40732900 | **LRRK2** | ENSG00000188906 | C | A | 0/1 |
| rs34637584 | 12 | 40734202 | **LRRK2** | ENSG00000188906 | G | A | 0/0 |
| rs10506155 | 12 | 40735939 | **LRRK2** | ENSG00000188906 | G | A | 0/1 |
| rs10878405 | 12 | 40742254 | **LRRK2** | ENSG00000188906 | G | A | 0/1 |
| rs11176143 | 12 | 40742363 | **LRRK2** | ENSG00000188906 | G | A | 0/0 |
| rs7303525 | 12 | 40744775 | **LRRK2** | ENSG00000188906 | A | C | 0/1 |
| rs7132187 | 12 | 40744808 | **LRRK2** | ENSG00000188906 | G | A | 0/1 |
| rs34778348 | 12 | 40757328 | **LRRK2** | ENSG00000188906 | G | A | 0/0 |
| rs7210728 | 17 | 43968415 | **MAPT** | ENSG00000186868 | G | A | 0/0 |
| rs11079727 | 17 | 43976813 | **MAPT** | ENSG00000186868 | C | A | 0/0 |
| rs3785879 | 17 | 43985636 | **MAPT** | ENSG00000186868 | C | A | 0/1 |
| rs1467967 | 17 | 43986179 | **MAPT** | ENSG00000186868 | G | A | 0/1 |
| rs63750959 | 17 | 44039717 | **MAPT** | ENSG00000186868 | G | A | 0/0 |
| rs754593 | 17 | 44054696 | **MAPT** | ENSG00000186868 | G | A | 1/1 |
| rs2435207 | 17 | 44058928 | **MAPT** | ENSG00000186868 | G | A | 0/0 |
| rs76375268 | 17 | 44060807 | **MAPT** | ENSG00000186868 | G | A | 0/0 |
| rs62063786 | 17 | 44061023 | **MAPT** | ENSG00000186868 | G | A | 0/0 |
| rs143624519 | 17 | 44068850 | **MAPT** | ENSG00000186868 | G | A | 0/0 |
| rs1052551 | 17 | 44068924 | **MAPT** | ENSG00000186868 | G | A | 0/0 |
| rs7220988 | 17 | 44109474 | **MAPT** | ENSG00000186868 | G | A | 0/0 |
| rs1078997 | 17 | 44109843 | **MAPT** | ENSG00000186868 | A | G | 0/1 |
| rs645419 | 11 | 102716321 | MMP3 | ENSG00000149968 | A | G | 1/1 |
| rs35231465 | 11 | 102584135 | MMP8 | ENSG00000118113 | G | A | 0/0 |
| rs3740938 | 11 | 102587062 | MMP8 | ENSG00000118113 | G | A | 0/1 |
| rs12941497 | 17 | 38255594 | **NR1D1** | ENSG00000126368 | G | A | 0/0 |
| rs2071570 | 17 | 38257090 | **NR1D1** | ENSG00000126368 | C | A | 0/0 |
| rs151227 | 16 | 28549508 | NUPR1 | ENSG00000176046 | G | A | 0/0 |
| rs63750592 | 14 | 73637521 | **PSEN1** | ENSG00000080815 | G | A | 0/0 |
| rs689470 | 1 | 186641058 | PTGS2 | ENSG00000073756 | G | A | 0/0 |
| rs689467 | 1 | 186641273 | PTGS2 | ENSG00000073756 | A | C | 0/1 |
| rs5275 | 1 | 186643058 | PTGS2 | ENSG00000073756 | A | G | 0/1 |
| rs16843670 | 1 | 198650006 | PTPRC | ENSG00000081237 | G | A | 0/0 |
| rs10494783 | 1 | 198663661 | PTPRC | ENSG00000081237 | G | A | 0/0 |
| rs2274367 | 1 | 198677342 | PTPRC | ENSG00000081237 | G | A | 0/0 |
| rs61746143 | 7 | 128843396 | **SMO** | ENSG00000128602 | G | A | 0/0 |
| rs147491841 | 7 | 128843411 | **SMO** | ENSG00000128602 | G | A | 0/0 |
| rs112783338 | 7 | 128852155 | **SMO** | ENSG00000128602 | C | A | 0/0 |
| rs1061285 | 7 | 128853308 | **SMO** | ENSG00000128602 | C | A | 0/0 |
| rs3775439 | 4 | 90709741 | SNCA | ENSG00000145335 | G | A | 0/0 |
| rs12502363 | 4 | 90713064 | SNCA | ENSG00000145335 | G | A | 0/0 |
| rs1812923 | 4 | 90739539 | SNCA | ENSG00000145335 | C | A | 0/0 |
| rs2301134 | 4 | 90758945 | SNCA | ENSG00000145335 | A | G | 0/0 |
| rs141647540 | 4 | 68424562 | STAP1 | ENSG00000035720 | G | A | 0/0 |
| rs3775866 | 4 | 68444180 | STAP1 | ENSG00000035720 | G | A | 0/1 |
| rs2242330 | 4 | 68447249 | STAP1 | ENSG00000035720 | A | G | 0/1 |
| rs11938228 | 4 | 154621946 | **TLR2** | ENSG00000137462 | C | A | 1/1 |
| rs5743703 | 4 | 154625795 | **TLR2** | ENSG00000137462 | G | A | 0/0 |
| rs5743704 | 4 | 154625951 | **TLR2** | ENSG00000137462 | C | A | 0/0 |
| rs5743305 | 4 | 186989333 | TLR3 | ENSG00000164342 | T | A | 0/1 |
| rs5743309 | 4 | 186990345 | TLR3 | ENSG00000164342 | C | A | 0/0 |
| rs11721827 | 4 | 186991137 | TLR3 | ENSG00000164342 | A | C | 0/0 |
| rs13126816 | 4 | 186994178 | TLR3 | ENSG00000164342 | G | A | 0/1 |
| rs3775296 | 4 | 186997767 | TLR3 | ENSG00000164342 | C | A | 0/1 |
| rs3775290 | 4 | 187004217 | TLR3 | ENSG00000164342 | C | A | 0/1 |
| rs10025405 | 4 | 187006806 | TLR3 | ENSG00000164342 | A | G | 0/1 |
| rs5743820 | 4 | 38828828 | TLR6 | ENSG00000174130 | G | A | 0/0 |
| rs5743813 | 4 | 38830062 | TLR6 | ENSG00000174130 | G | A | 0/0 |
| rs5743808 | 4 | 38830736 | TLR6 | ENSG00000174130 | A | G | 0/0 |
| rs179013 | X | 12901471 | TLR7 | ENSG00000196664 | G | A | 0/1 |
| rs179012 | X | 12901562 | TLR7 | ENSG00000196664 | G | A | 0/1 |
| rs179008 | X | 12903659 | TLR7 | ENSG00000196664 | A | T | 0/1 |
| rs864058 | X | 12906030 | TLR7 | ENSG00000196664 | G | A | 0/0 |
| rs3764880 | X | 12924826 | TLR8 | ENSG00000101916 | A | G | 0/1 |
| rs5744081 | X | 12938471 | TLR8 | ENSG00000101916 | C | A | 0/0 |
| rs2407992 | X | 12939112 | TLR8 | ENSG00000101916 | G | C | 0/1 |
| rs3747414 | X | 12939412 | TLR8 | ENSG00000101916 | C | A | 0/1 |
| rs522807 | 1 | 12224239 | **TNFRSF1B** | ENSG00000028137 | C | A | 0/1 |
| rs945439 | 1 | 12248942 | **TNFRSF1B** | ENSG00000028137 | A | G | 0/0 |
| rs683240 | 1 | 12249829 | **TNFRSF1B** | ENSG00000028137 | A | G | 0/0 |
| rs5746026 | 1 | 12253062 | **TNFRSF1B** | ENSG00000028137 | G | A | 0/0 |
| rs1061624 | 1 | 12267265 | **TNFRSF1B** | ENSG00000028137 | A | G | 0/1 |
| rs138355759 | 6 | 41126619 | **TREM2** | ENSG00000095970 | G | A | 0/0 |
| rs2234255 | 6 | 41127543 | **TREM2** | ENSG00000095970 | G | A | 0/0 |
| rs7748513 | 6 | 41127972 | **TREM2** | ENSG00000095970 | A | G | 0/0 |
| rs4790522 | 17 | 3469853 | TRPV1 | ENSG00000196689 | A | C | 1/1 |
| rs16953163 | 17 | 3471239 | TRPV1 | ENSG00000196689 | A | G | 0/1 |
| rs3826503 | 17 | 3474660 | TRPV1 | ENSG00000196689 | G | A | 0/1 |
| rs150908 | 17 | 3484368 | TRPV1 | ENSG00000196689 | G | A | 1/1 |
| rs224534 | 17 | 3486702 | TRPV1 | ENSG00000196689 | G | A | 1/1 |
| rs222745 | 17 | 3488871 | TRPV1 | ENSG00000196689 | G | A | 0/0 |
| rs222747 | 17 | 3493200 | TRPV1 | ENSG00000196689 | C | G | 1/1 |
| rs222749 | 17 | 3495374 | TRPV1 | ENSG00000196689 | G | A | 0/0 |
